# Supplementary material for: Long-term assessment of ecosystem services at ecological restoration sites using Landsat time series
Source: PLoS One. 2021 Jun 23;16(6):e0243020. doi: 10.1371/journal.pone.0243020 (PMC8221468; doi:10.1371/journal.pone.0243020)
Supplement: S1 File — (DOCX) [file pone.0243020.s001.docx]

**S1 File**

S1 Fig. Annual cumulative rainfall for 1990-2018. Dashed line indicates the average rainfall for the period (327 mm). Source: Data averaged for 16 rain stations located in the Baviaanskloof catchment [1].


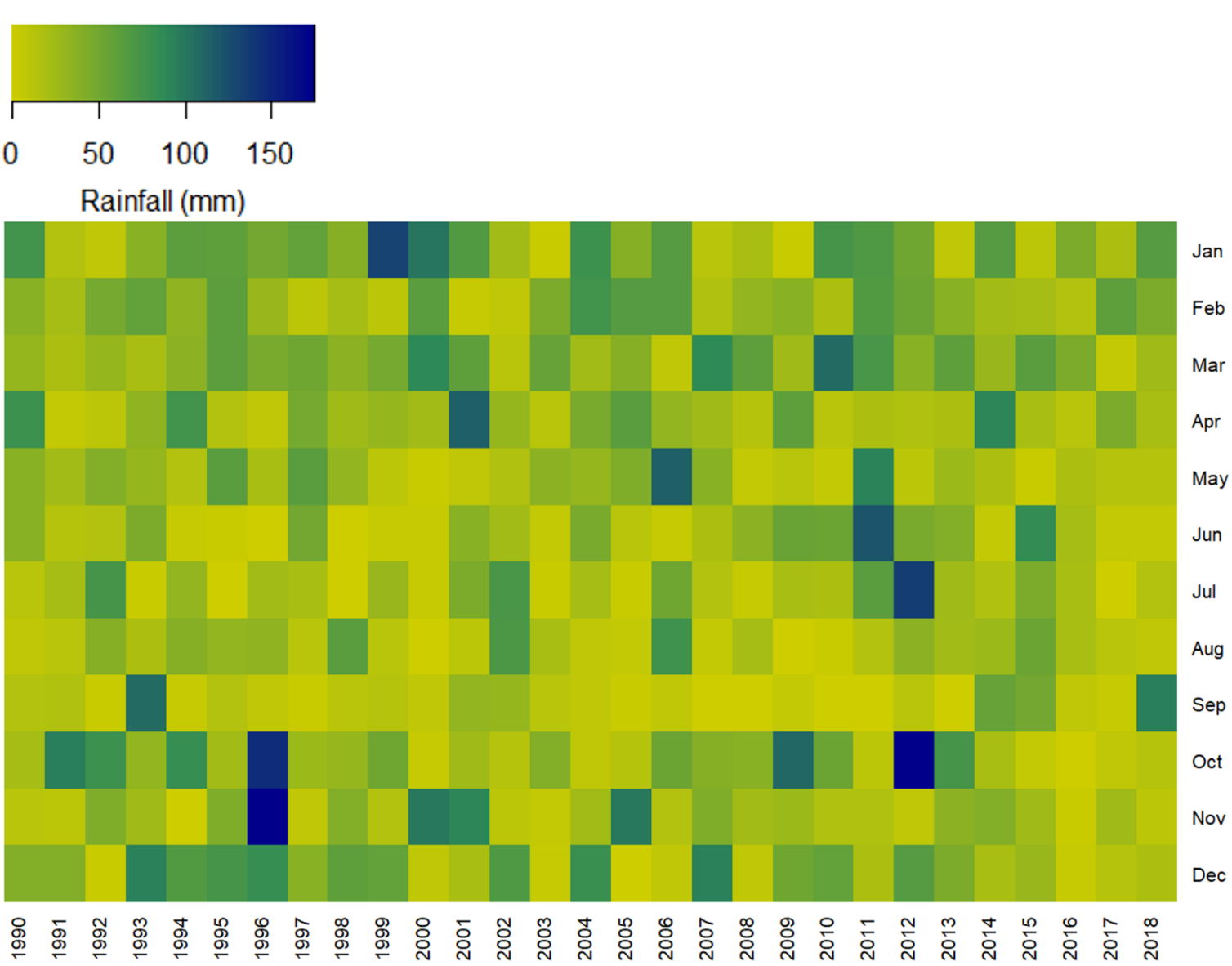


S2 Fig. Heatmap of monthly rainfall (mm) for period 1990- 2018. Source: Data averaged from 16 rain stations located in the Baviaanskloof catchment [1].

S1 Table. Landsat images used for ISODATA clustering.

| **Satellite** | **Date** |
| --- | --- |
| Landsat 5 | 26-Feb-89 |
|  | 02-Jun-89 |
|  | 18-Jun-89 |
|  | 20-Jul-89 |
|  | 05-Aug-89 |
|  | 21-Aug-89 |
|  | 01-Mar-90 |
|  | 17-Mar-90 |
|  | 25-Sep-90 |
|  | 27-Oct-90 |

S2 Table. Landsat images used for BACI analyses for the period before and after.

| **Invention Period** | **Intervention** | **Ecosystem service** | | | | | |
| --- | --- | --- | --- | --- | --- | --- | --- |
|  |  | **Erosion prevention** | | **Provision of forage** | | **Presence native species** | |
|  |  | **Date** | **Satellite and sensor** | **Date** | **Satellite and sensor** | **Date** | **Satellite and sensor** |
| Before | Livestock exclusion | 11/12/1989 | Landsat 5 TM | 20/07/1989 | Landsat 5 TM | 02/06/1989 | Landsat 5 TM |
|  |  | 01/03/1990 |  | 17/03/1990 |  | 17/03/1990 |  |
|  | Livestock exclusion + Revegetation | 11/12/1989 |  | 20/07/1989 |  | 02/06/1989 |  |
|  |  | 01/03/1990 |  | 17/03/1990 |  | 17/03/1990 |  |
|  | Revegetation 2010 | 09/04/2007 | Landsat 7 ETM+ | 27/05/2007 | Landsat 7 ETM+ | 09/04/2007 | Landsat 7 ETM+ |
|  |  | 22/01/2008 |  | 30/06/2008 |  | 22/01/2008 |  |
|  |  | 08/05/2009 | Landsat 8 OLI | 17/06/2009 |  | 08/05/2009 | Landsat 5 TM |
|  | Revegetation 2011 | 22/01/2008 | Landsat 7 ETM+ | 30/06/2008 | Landsat 7 ETM+ | 22/01/2008 | Landsat 7 ETM+ |
|  |  | 08/05/2009 | Landsat 5 TM | 17/06/2009 |  | 08/05/2009 | Landsat 5 TM |
|  |  | 22/07/2010 | Landsat 7 ETM+ | 20/06/2010 |  | 20/06/2010 | Landsat 7 ETM+ |
|  | Revegetation 2012 | 08/05/2009 | Landsat 5 TM | 17/06/2009 | Landsat 7 ETM+ | 08/05/2009 | Landsat 5 TM |
|  |  | 22/07/2010 | Landsat 7 ETM+ | 20/06/2010 |  | 20/06/2010 | Landsat 7 ETM+ |
|  |  | 09/07/2011 |  | 10/08/2011 |  | 10/08/2011 |  |
|  | Revegetation 2013 | 22/07/2010 | Landsat 7 ETM+ | 20/06/2010 | Landsat 7 ETM+ | 20/06/2010 | Landsat 7 ETM+ |
|  |  | 09/07/2011 |  | 10/08/2011 |  | 10/08/2011 |  |
|  |  | 29/09/2012 |  | 28/08/2012 |  | 29/09/2012 |  |
|  | Revegetation 2014 | 09/07/2011 | Landsat 7 ETM+ | 10/08/2011 | Landsat 7 ETM+ | 10/08/2011 | Landsat 7 ETM+ |
|  |  | 29/09/2012 |  | 28/08/2012 |  | 29/09/2012 |  |
|  |  | 28/06/2013 |  | 06/07/2013 | Landsat 8 OLI | 20/06/2013 | Landsat 8 OLI |
|  | Revegetation 2015 | 29/09/2012 | Landsat 7 ETM+ | 28/08/2012 | Landsat 7 ETM+ | 29/09/2012 | Landsat 7 ETM+ |
|  |  | 28/06/2013 |  | 06/07/2013 | Landsat 8 OLI | 20/06/2013 | Landsat 8 OLI |
|  |  | 23/06/2014 | Landsat 8 OLI | 23/06/2014 |  | 20/04/2014 |  |
| After | All | 30/05/2017 | Landsat 8 OLI | 14/05/2017 | Landsat 8 OLI | 14/05/2017 | Landsat 8 OLI |
|  |  | 18/04/2018 |  | 15/04/2018 |  | 15/04/2018 |  |
|  |  | 20/05/2019 |  | 20/05/2019 |  | 20/05/2019 |  |


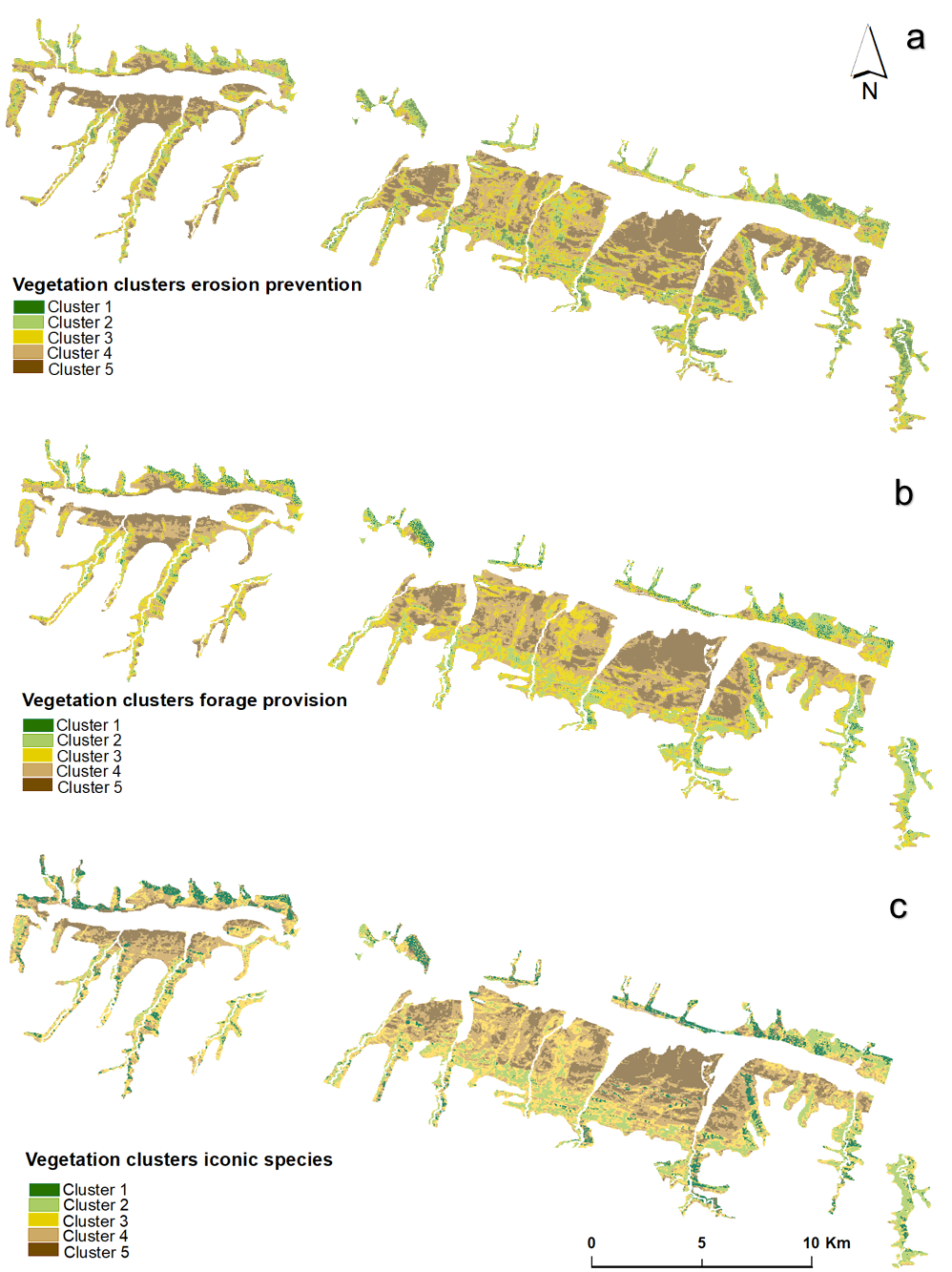


S3 Fig. Vegetation clusters resulting from ISODATA classification before the interventions occurred for a) erosion prevention, b) forage provision and c) presence of iconic species.


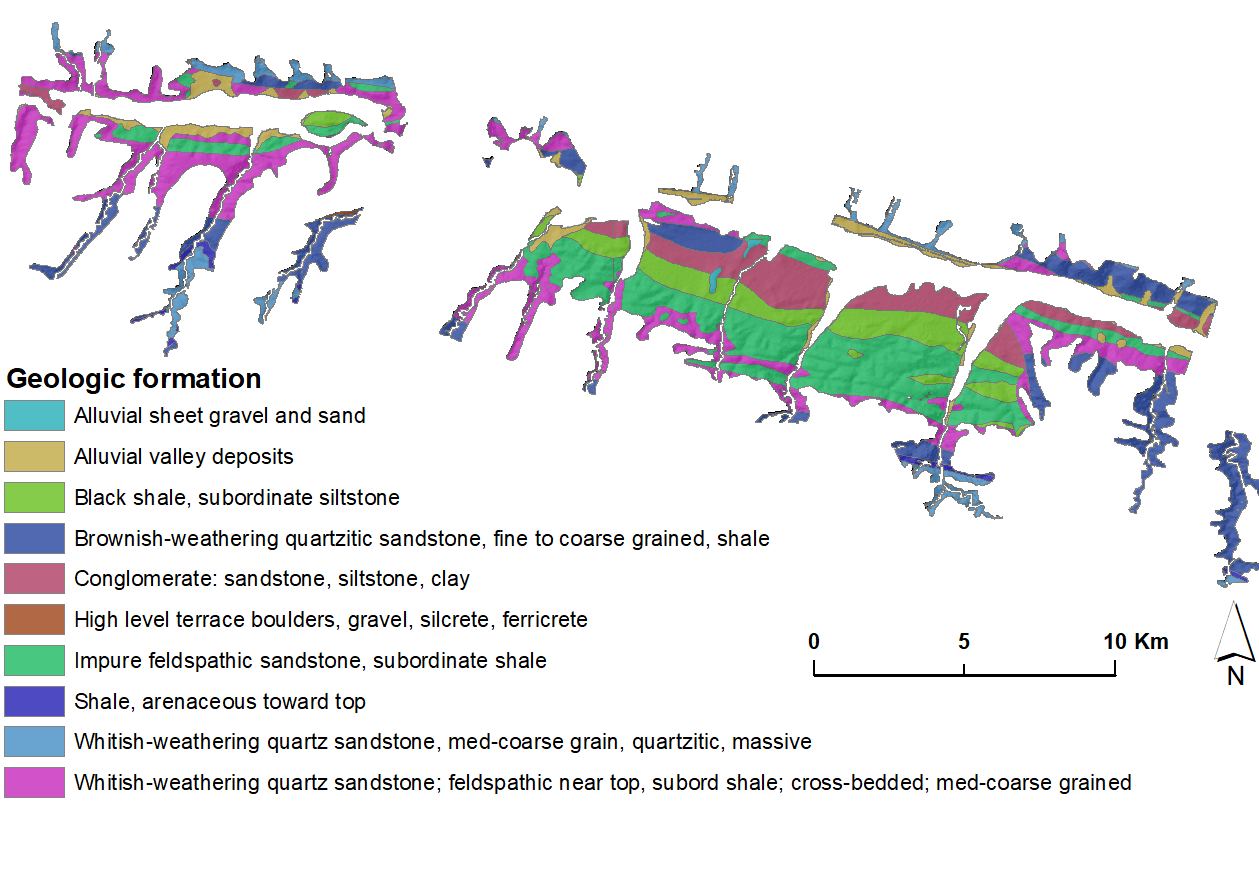


S4 Fig. Soil parent material classes present in the study area. Source: South African Council for Geoscience


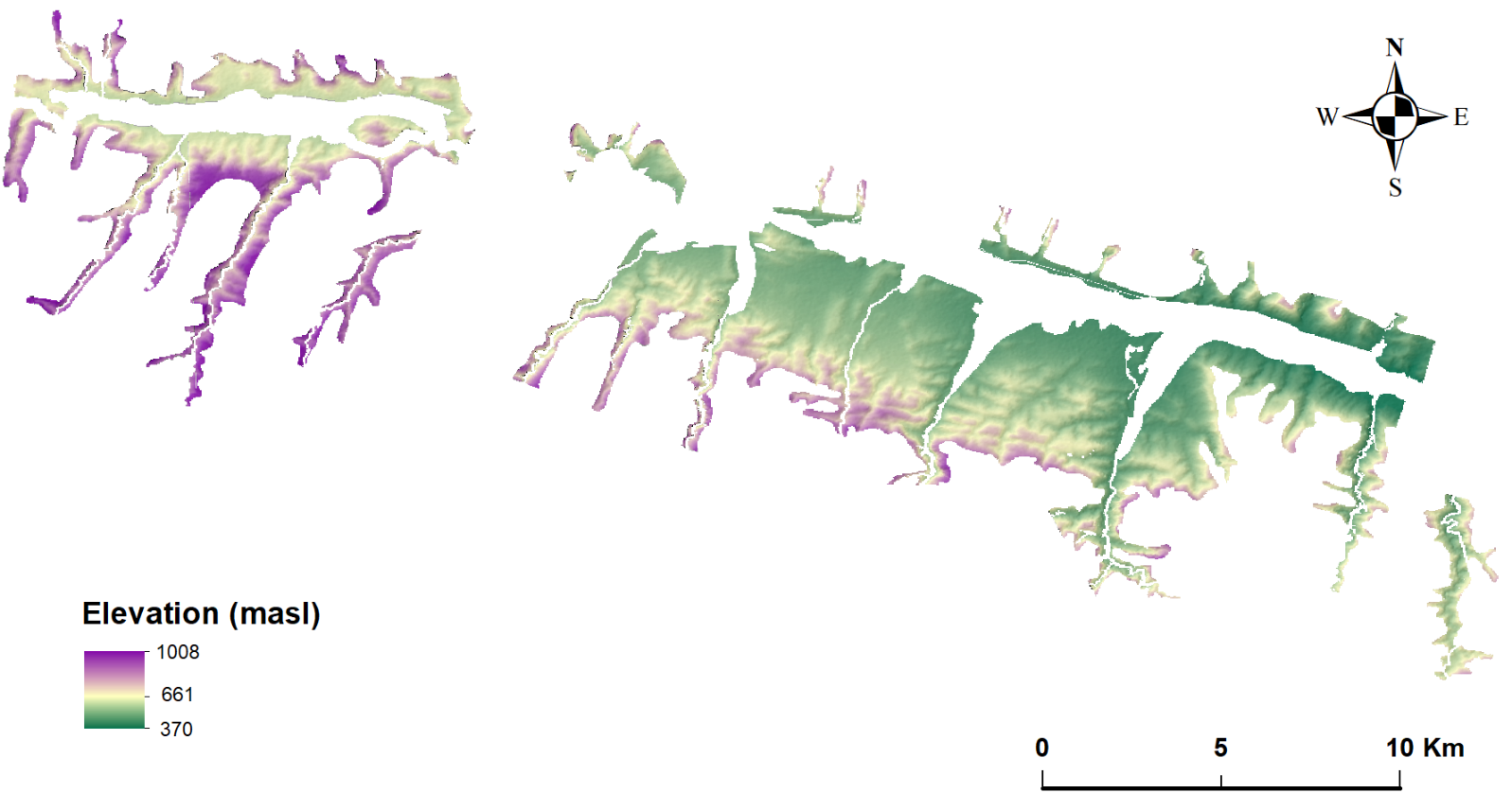


S5 Fig. Elevation map for the study area derived from the 12.5 m resolution ALOS PALSAR derived DEM from the Geophysical Institute of the University of Alaska Fairbanks [2].


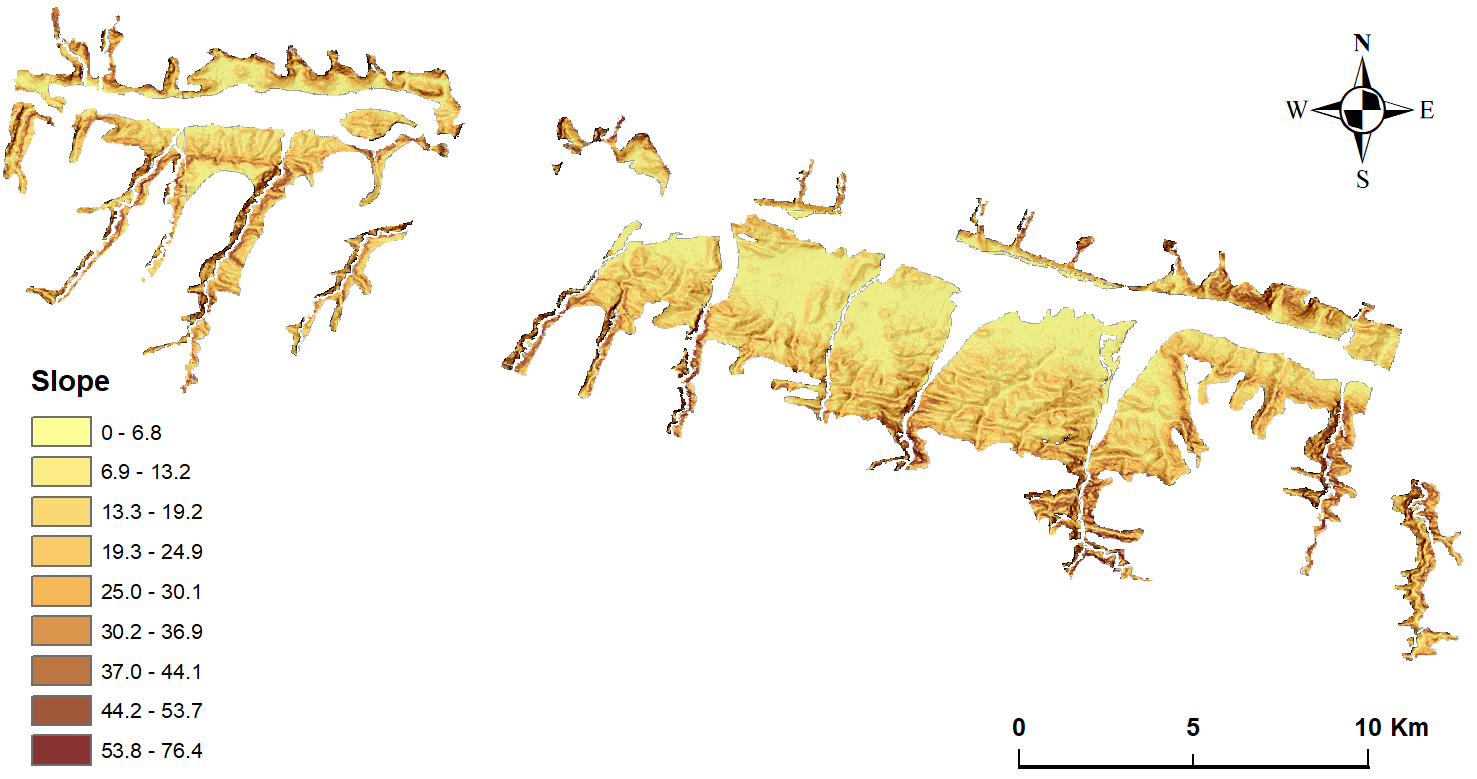


S6 Fig. Slope map for the study area derived from the 12.5 m resolution ALOS PALSAR DEM from the Geophysical Institute of the University of Alaska Fairbanks [2]. Shading indicates topographic relief.


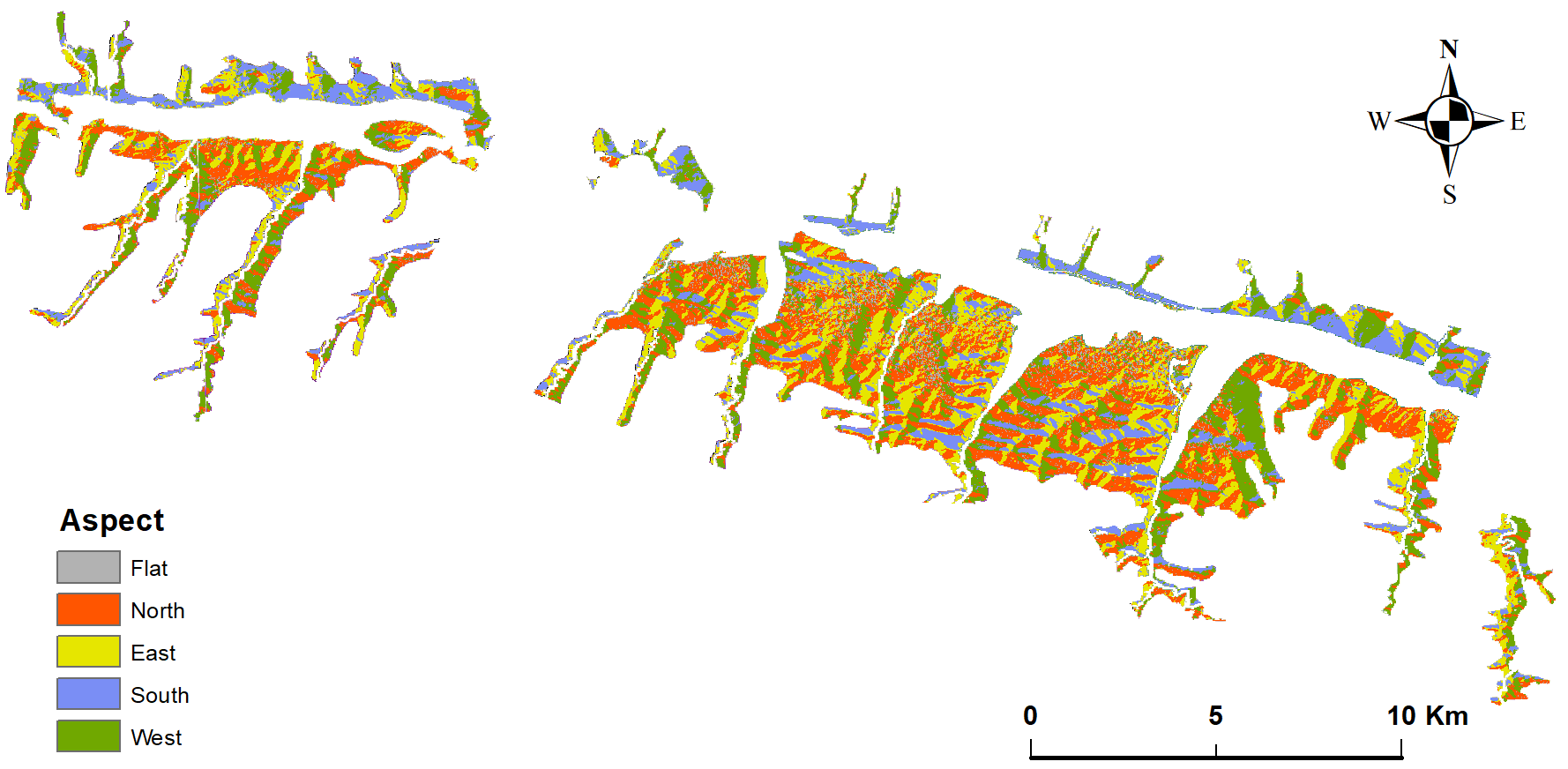


S7 Fig. Aspect map for the study area derived from the 12.5 m resolution ALOS PALSAR DEM from the Geophysical Institute of the University of Alaska Fairbanks [2]


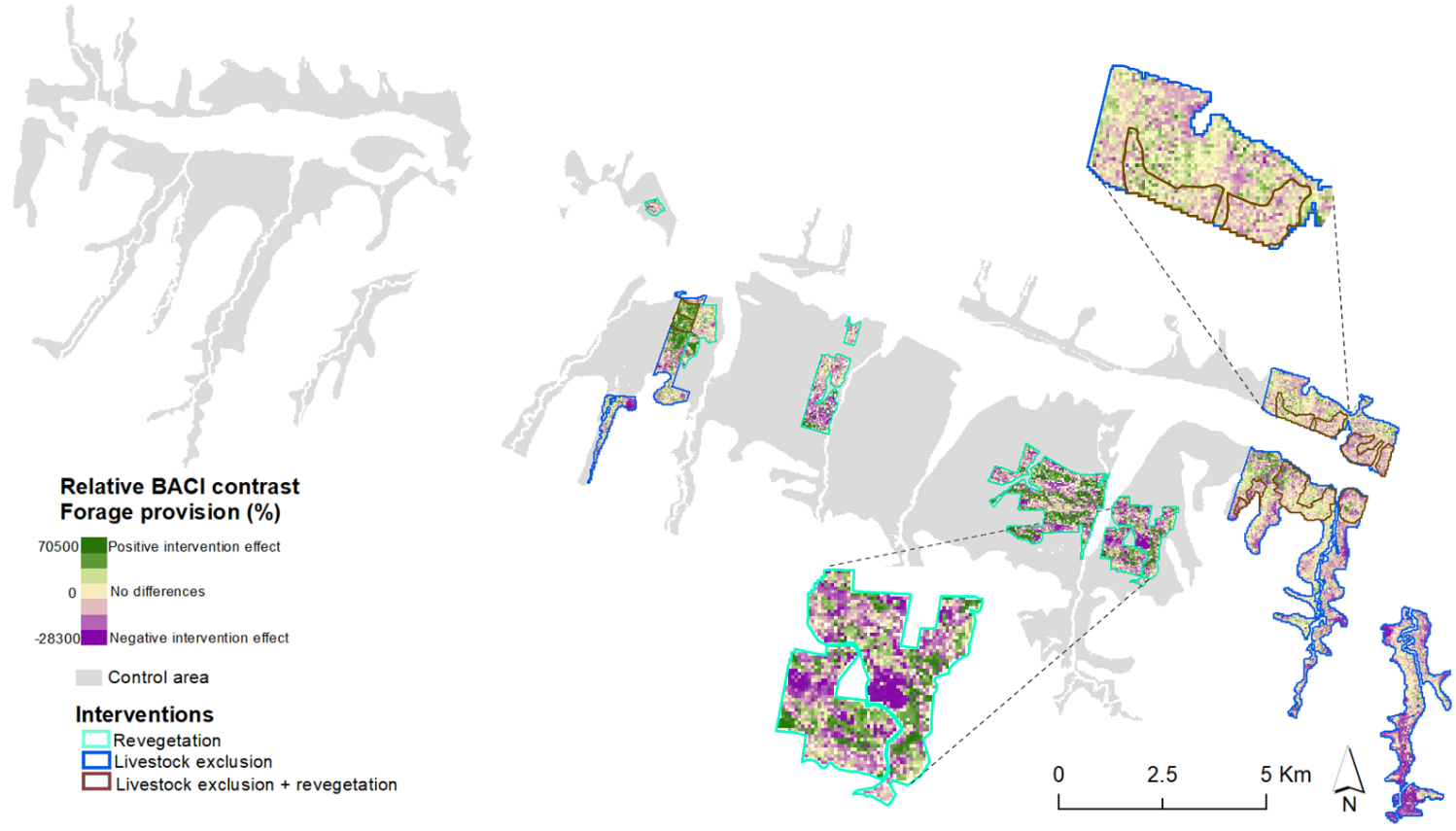


S8 Fig. Relative BACI contrast at pixel level to assess the effect of intervention on provision of forage (based on NBR index). The thicket and shrubland area used to for selecting control sites is indicated in grey.


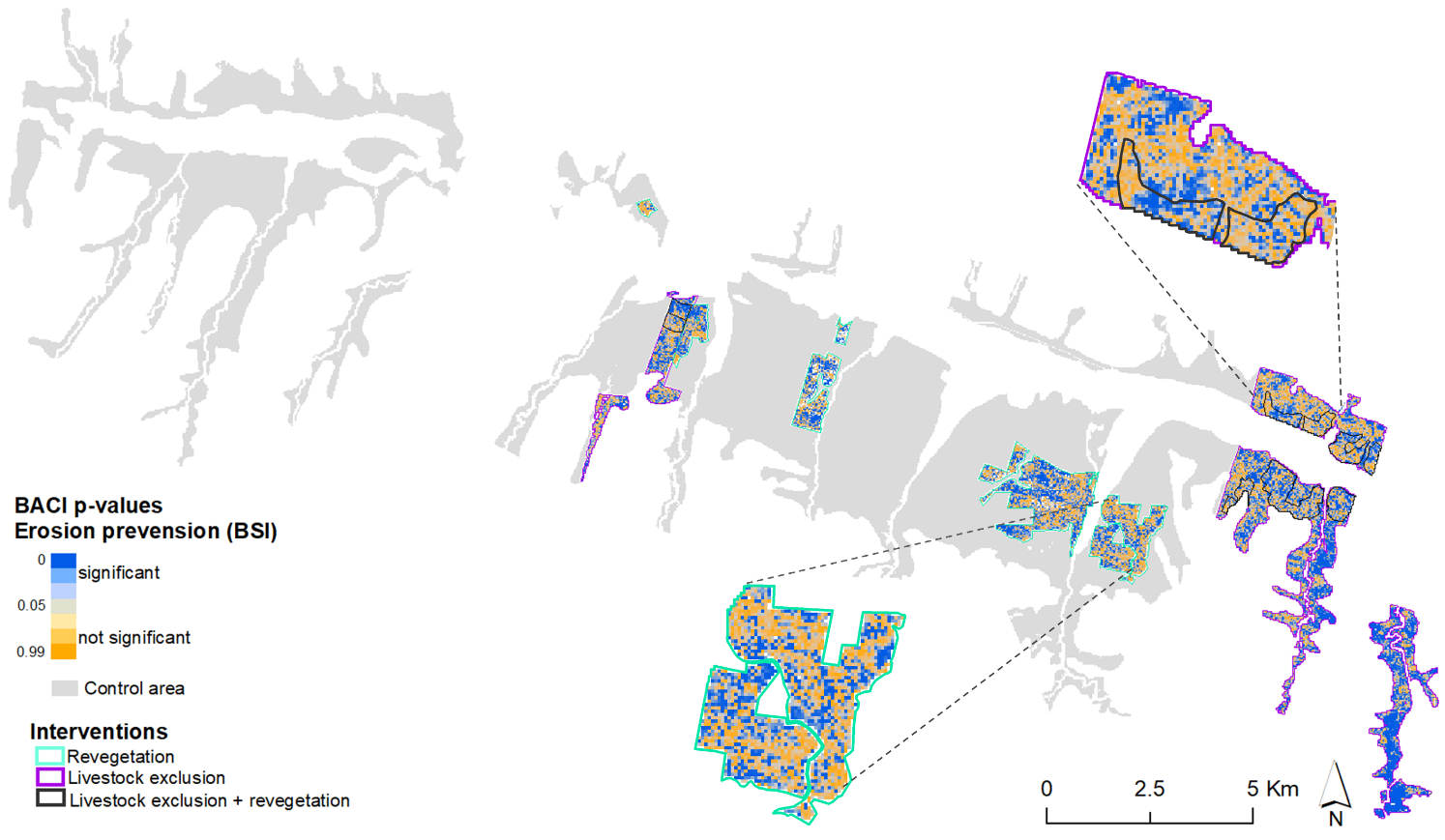


S9 Fig. Spatial distribution of p-values of BACI contrast at pixel-level to assess the effect of each restoration intervention on erosion prevention (based in the BSI index). The thicket and shrubland area used to for selecting control sites is indicated in grey.

S3 Table. Comparison of BACI contrast for each intervention between terrain aspect and ecosystem service using the Kruskal-Wallis test (Games-Howell post-hoc). IQR = interquartile range.

| Landsat index | Represented ecosystem service | Cluster | Hodges-Lehmann estimator | IQR | Post hoc* |
| --- | --- | --- | --- | --- | --- |
| BSI | Erosion prevention | North | 0.01 | 0.06 | a |
|  |  | South | 0.02 | 0.09 | b |
|  |  | East | 0.01 | 0.08 | c |
|  |  | West | 0.02 | 0.09 | b |
|  |  | Flat | 0.00 | 0.05 | a |
| NBR | Provision of forage | North | 0.00 | 0.09 | ab |
|  |  | South | 0.03 | 0.14 | c |
|  |  | East | 0.00 | 0.10 | a |
|  |  | West | 0.04 | 0.16 | c |
|  |  | Flat | 0.01 | 0.08 | b |
| MSAVI | Presence of iconic species | North | 0.01 | 0.08 | a |
|  |  | South | 0.01 | 0.10 | b |
|  |  | East | 0.02 | 0.08 | c |
|  |  | West | 0.01 | 0.11 | bd |
|  |  | Flat | 0.01 | 0.08 | abd |

*different letters indicate significant differences of Games-Howell post-hoc at p-value < 0.05

S4 Table. Comparison of BACI contrast between soil parent material classes for each intervention and ecosystem service using the Kruskal-Wallis test (Games-Howell post-hoc). IQR = interquartile range.

| Landsat index | Represented ecosystem service | Geologic formation | Hodges-Lehmann estimator | IQR | Post Hoc* |
| --- | --- | --- | --- | --- | --- |
| BSI | Erosion prevention | Brownish quartzitic sandstone | 0.04 | 0.11 | a |
|  |  | Feldspathic sandstone | 0.00 | 0.06 | b |
|  |  | Shale | 0.00 | 0.05 | b |
|  |  | Enon conglomerate | 0.00 | 0.06 | b |
|  |  | Whitish quartzitic sandstone | 0.01 | 0.07 | c |
| NBR | Provision of forage | Brownish quartzitic sandstone | 0.04 | 0.13 | a |
|  |  | Feldspathic sandstone | -0.02 | 0.10 | b |
|  |  | Shale | 0.00 | 0.09 | c |
|  |  | Enon conglomerate | 0.00 | 0.10 | c |
|  |  | Whitish quartzitic sandstone | -0.01 | 0.06 | b |
| MSAVI | Presence of native species | Brownish quartzitic sandstone | 0.03 | 0.11 | a |
|  |  | Feldspathic sandstone | 0.00 | 0.08 | b |
|  |  | Shale | 0.01 | 0.08 | c |
|  |  | Enon conglomerate | 0.02 | 0.08 | ac |
|  |  | Whitish quartzitic sandstone | 0.01 | 0.07 | ac |

*different letters indicate significant differences of Games-Howell post-hoc at p-value < 0.05

S5 Table. Coefficient of determination for different regression models tested between BACI contrast with elevation and slope

| Landsat index | Represented ecosystem service | Slope | | Elevation | | |
| --- | --- | --- | --- | --- | --- | --- |
|  |  | R^2^ linear | R^2^ polynomial* | R^2^ linear | R^2^ exponential | R^2^ polynomial* |
| BSI | Erosion prevention | 0.03 | 0.04 | < 0.01 | < 0.01 | 0.05 |
| NBR | Provision of forage | 0.01 | 0.02 | 0.03 | 0.02 | 0.03 |
| MSAVI | Presence of iconic species | 0.02 | 0.03 | < 0.01 | < 0.01 | < 0.01 |

*2^nd^ degree polynomial

S6 Table. Explorative analysis. Differences between BACI contrast and p-values for forage provision in pixels under revegetation using 20 and 100 controls.

| Pixel number | Cluster number | BACI 20 controls | BACI 100 controls | p-value 20 controls | p-value 100 controls |
| --- | --- | --- | --- | --- | --- |
| 1 | 3 | -0.051 | -0.057 | 0.000 | 0.000 |
| 2 | 3 | -0.049 | -0.052 | 0.000 | 0.000 |
| 3 | 3 | -0.063 | -0.050 | 0.000 | 0.000 |
| 4 | 3 | -0.062 | -0.071 | 0.000 | 0.000 |
| 5 | 3 | -0.055 | -0.051 | 0.000 | 0.000 |
| 6 | 3 | 0.050 | 0.036 | 0.017 | 0.000 |
| 7 | 3 | 0.052 | 0.038 | 0.011 | 0.000 |
| 8 | 3 | 0.075 | 0.052 | 0.001 | 0.000 |
| 9 | 3 | 0.082 | 0.065 | 0.000 | 0.000 |
| 10 | 3 | 0.036 | 0.030 | 0.008 | 0.000 |
| 11 | 3 | 0.004 | 0.000 | 0.002 | 0.000 |
| 12 | 3 | -0.031 | -0.018 | 0.000 | 0.000 |
| 13 | 3 | -0.036 | -0.044 | 0.002 | 0.000 |
| 14 | 3 | -0.034 | -0.031 | 0.001 | 0.000 |
| 15 | 3 | -0.031 | -0.045 | 0.132 | 0.000 |
| 16 | 3 | -0.041 | -0.054 | 0.042 | 0.000 |
| 17 | 3 | -0.059 | -0.081 | 0.006 | 0.000 |
| 18 | 3 | -0.055 | -0.072 | 0.012 | 0.000 |
| 19 | 3 | -0.056 | -0.062 | 0.000 | 0.000 |
| 20 | 3 | -0.064 | -0.067 | 0.000 | 0.000 |
| 21 | 3 | -0.050 | -0.038 | 0.000 | 0.000 |
| 22 | 3 | -0.042 | -0.050 | 0.001 | 0.000 |
| 23 | 3 | -0.054 | -0.051 | 0.000 | 0.000 |
| 24 | 3 | 0.075 | 0.061 | 0.001 | 0.000 |
| 25 | 3 | 0.091 | 0.077 | 0.000 | 0.000 |
| 26 | 3 | 0.116 | 0.094 | 0.000 | 0.000 |
| 27 | 3 | 0.111 | 0.095 | 0.000 | 0.000 |
| 28 | 3 | 0.033 | 0.027 | 0.007 | 0.000 |
| 29 | 3 | 0.010 | 0.007 | 0.004 | 0.000 |
| 30 | 3 | -0.018 | -0.005 | 0.001 | 0.000 |
| 31 | 3 | -0.012 | -0.020 | 0.015 | 0.000 |
| 32 | 3 | -0.014 | -0.011 | 0.005 | 0.000 |
| 33 | 3 | -0.014 | -0.028 | 0.486 | 0.001 |
| 34 | 3 | -0.022 | -0.036 | 0.261 | 0.000 |
| 35 | 3 | -0.048 | -0.070 | 0.024 | 0.000 |
| 36 | 3 | -0.026 | -0.043 | 0.219 | 0.000 |
| 37 | 3 | -0.035 | -0.041 | 0.000 | 0.000 |
| 38 | 3 | -0.023 | -0.026 | 0.000 | 0.000 |
| 39 | 3 | -0.060 | -0.048 | 0.000 | 0.000 |
| 40 | 3 | -0.069 | -0.077 | 0.000 | 0.000 |
| 41 | 3 | -0.043 | -0.039 | 0.000 | 0.000 |
| 42 | 3 | 0.024 | 0.010 | 0.243 | 0.300 |
| 43 | 3 | 0.052 | 0.038 | 0.011 | 0.000 |
| 44 | 3 | 0.059 | 0.037 | 0.006 | 0.000 |
| 45 | 3 | 0.054 | 0.038 | 0.013 | 0.000 |
| 46 | 3 | 0.051 | 0.045 | 0.021 | 0.000 |
| 47 | 3 | 0.038 | 0.035 | 0.023 | 0.000 |
| 48 | 3 | -0.007 | 0.005 | 0.003 | 0.000 |
| 49 | 3 | -0.010 | -0.018 | 0.017 | 0.000 |
| 50 | 3 | -0.006 | -0.002 | 0.010 | 0.000 |
| 51 | 3 | -0.012 | -0.026 | 0.561 | 0.003 |
| 52 | 3 | -0.006 | -0.020 | 0.748 | 0.019 |
| 53 | 3 | -0.023 | -0.045 | 0.268 | 0.000 |
| 54 | 3 | -0.015 | -0.032 | 0.464 | 0.001 |
| 55 | 3 | -0.057 | -0.063 | 0.000 | 0.000 |
| 56 | 3 | -0.092 | -0.095 | 0.000 | 0.000 |
| 57 | 3 | -0.060 | -0.047 | 0.000 | 0.000 |
| 58 | 3 | -0.087 | -0.095 | 0.000 | 0.000 |
| 59 | 3 | -0.053 | -0.050 | 0.000 | 0.000 |
| 60 | 3 | 0.048 | 0.034 | 0.021 | 0.000 |
| 61 | 3 | 0.041 | 0.028 | 0.041 | 0.002 |
| 62 | 3 | 0.065 | 0.043 | 0.003 | 0.000 |
| 63 | 3 | 0.062 | 0.045 | 0.005 | 0.000 |
| 64 | 3 | 0.058 | 0.051 | 0.031 | 0.002 |
| 65 | 3 | 0.015 | 0.012 | 0.005 | 0.000 |
| 66 | 3 | -0.005 | 0.008 | 0.003 | 0.001 |
| 67 | 3 | 0.000 | -0.009 | 0.033 | 0.000 |
| 68 | 3 | 0.033 | 0.037 | 0.206 | 0.099 |
| 69 | 3 | 0.010 | -0.004 | 0.636 | 0.568 |
| 70 | 3 | 0.003 | -0.010 | 0.863 | 0.213 |
| 71 | 3 | -0.017 | -0.039 | 0.417 | 0.000 |
| 72 | 3 | -0.001 | -0.018 | 0.965 | 0.056 |
| 73 | 4 | -0.036 | -0.034 | 0.001 | 0.000 |
| 74 | 4 | -0.024 | -0.022 | 0.005 | 0.000 |
| 75 | 4 | 0.008 | -0.001 | 0.060 | 0.000 |
| 76 | 4 | 0.023 | 0.019 | 0.340 | 0.001 |
| 77 | 4 | 0.025 | 0.027 | 0.023 | 0.000 |
| 78 | 4 | 0.035 | 0.040 | 0.003 | 0.000 |
| 79 | 4 | 0.006 | 0.014 | 0.586 | 0.008 |
| 80 | 4 | 0.025 | 0.034 | 0.030 | 0.000 |
| 81 | 4 | -0.014 | -0.012 | 0.008 | 0.000 |
| 82 | 4 | -0.024 | -0.022 | 0.005 | 0.000 |
| 83 | 4 | -0.057 | -0.054 | 0.000 | 0.000 |
| 84 | 4 | -0.021 | -0.030 | 0.003 | 0.000 |
| 85 | 4 | 0.024 | 0.021 | 0.389 | 0.003 |
| 86 | 4 | 0.009 | 0.012 | 0.391 | 0.023 |
| 87 | 4 | 0.002 | 0.007 | 0.888 | 0.188 |
| 88 | 4 | -0.040 | -0.032 | 0.000 | 0.000 |
| 89 | 4 | -0.030 | -0.020 | 0.010 | 0.000 |
| 90 | 4 | -0.026 | -0.025 | 0.002 | 0.000 |
| 91 | 4 | -0.033 | -0.031 | 0.002 | 0.000 |
| 92 | 4 | -0.030 | -0.039 | 0.001 | 0.000 |
| 93 | 4 | 0.007 | 0.004 | 0.083 | 0.013 |
| 94 | 4 | 0.042 | 0.045 | 0.000 | 0.000 |
| 95 | 4 | 0.046 | 0.051 | 0.000 | 0.000 |
| 96 | 4 | 0.027 | 0.036 | 0.010 | 0.000 |
| 97 | 4 | 0.020 | 0.029 | 0.084 | 0.000 |
| 98 | 4 | -0.020 | -0.018 | 0.004 | 0.000 |
| 99 | 4 | -0.046 | -0.044 | 0.000 | 0.000 |
| 100 | 4 | -0.056 | -0.053 | 0.000 | 0.000 |
| 101 | 4 | -0.005 | -0.015 | 0.017 | 0.000 |
| 102 | 4 | 0.006 | 0.003 | 0.071 | 0.000 |
| 103 | 4 | -0.040 | -0.037 | 0.000 | 0.000 |
| 104 | 4 | -0.052 | -0.046 | 0.000 | 0.000 |
| 105 | 4 | -0.080 | -0.071 | 0.000 | 0.000 |
| 106 | 4 | -0.073 | -0.064 | 0.000 | 0.000 |
| 107 | 4 | -0.025 | -0.023 | 0.002 | 0.000 |
| 108 | 4 | -0.019 | -0.029 | 0.004 | 0.000 |
| 109 | 4 | 0.002 | -0.001 | 0.048 | 0.000 |
| 110 | 4 | -0.015 | -0.012 | 0.174 | 0.019 |
| 111 | 4 | -0.004 | 0.001 | 0.696 | 0.887 |
| 112 | 4 | -0.023 | -0.014 | 0.030 | 0.006 |
| 113 | 4 | -0.025 | -0.016 | 0.026 | 0.003 |
| 114 | 4 | 0.023 | 0.024 | 0.225 | 0.010 |
| 115 | 4 | -0.003 | -0.001 | 0.053 | 0.000 |
| 116 | 4 | 0.006 | 0.008 | 0.023 | 0.002 |
| 117 | 4 | 0.017 | 0.007 | 0.126 | 0.000 |
| 118 | 4 | -0.003 | -0.006 | 0.026 | 0.000 |
| 119 | 4 | -0.094 | -0.092 | 0.000 | 0.000 |
| 120 | 4 | -0.100 | -0.095 | 0.000 | 0.000 |
| 121 | 4 | -0.114 | -0.106 | 0.000 | 0.000 |
| 122 | 4 | -0.132 | -0.123 | 0.000 | 0.000 |
| 123 | 4 | -0.011 | -0.009 | 0.011 | 0.000 |
| 124 | 4 | 0.018 | 0.009 | 0.141 | 0.000 |
| 125 | 4 | 0.039 | 0.035 | 0.914 | 0.067 |
| 126 | 4 | 0.030 | 0.033 | 0.007 | 0.000 |
| 127 | 4 | 0.047 | 0.052 | 0.000 | 0.000 |
| 128 | 4 | 0.009 | 0.018 | 0.362 | 0.001 |
| 129 | 4 | 0.013 | 0.023 | 0.247 | 0.001 |
| 130 | 4 | -0.010 | -0.008 | 0.012 | 0.000 |
| 131 | 4 | -0.028 | -0.026 | 0.003 | 0.000 |
| 132 | 4 | -0.045 | -0.042 | 0.000 | 0.000 |
| 133 | 4 | 0.016 | 0.007 | 0.117 | 0.000 |
| 134 | 4 | 0.003 | 0.000 | 0.051 | 0.000 |
| 135 | 4 | -0.061 | -0.058 | 0.000 | 0.000 |
| 136 | 4 | -0.071 | -0.066 | 0.000 | 0.000 |
| 137 | 4 | -0.091 | -0.083 | 0.000 | 0.000 |
| 138 | 4 | -0.099 | -0.089 | 0.000 | 0.000 |

***References***

1. WRC. Water Research Commission. Annual Report. Pretoria, South Africa: WRC; 2018.

2. Geophysical Institute of the University of Alaska Fairbanks. Alaska Satellite Facility (ASF) Data Portal [Internet]. 2018 [cited 2018 Jul 2]. Available from: https://vertex.daac.asf.alaska.edu/#
