# Supplementary figures and images for: Long-term assessment of ecosystem services at ecological restoration sites using Landsat time series
Source: PLoS One. 2021 Jun 23;16(6):e0243020. doi: 10.1371/journal.pone.0243020 (PMC8221468; doi:10.1371/journal.pone.0243020)

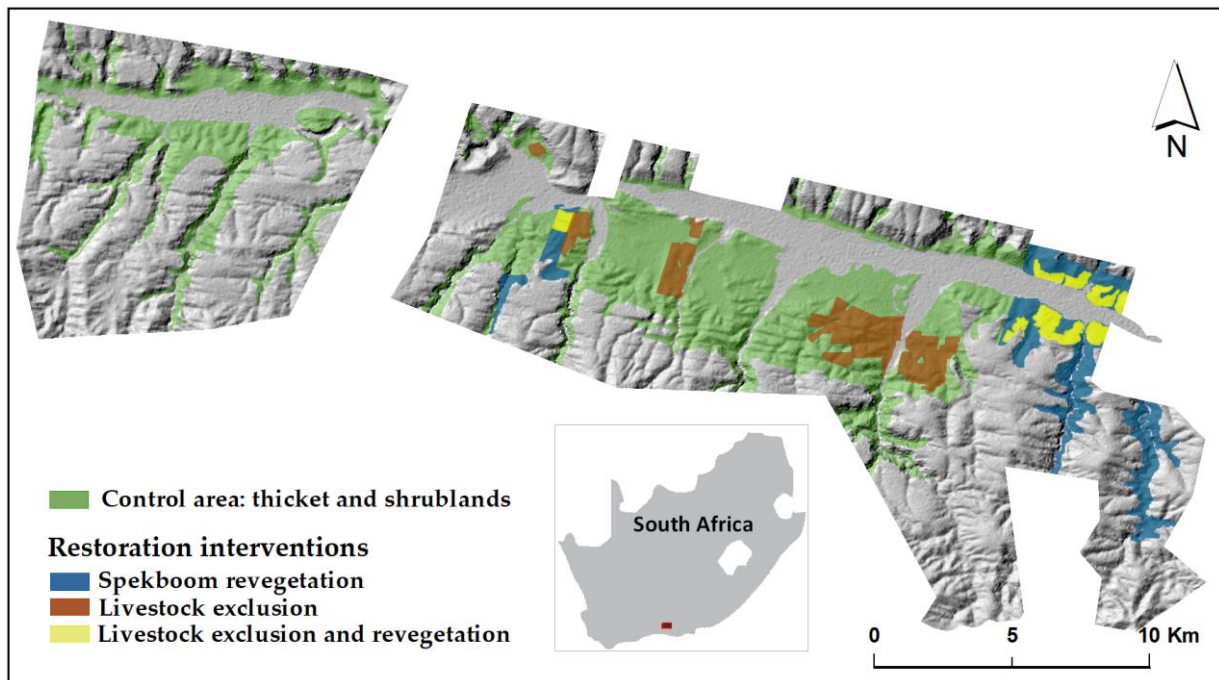

Fig 1.

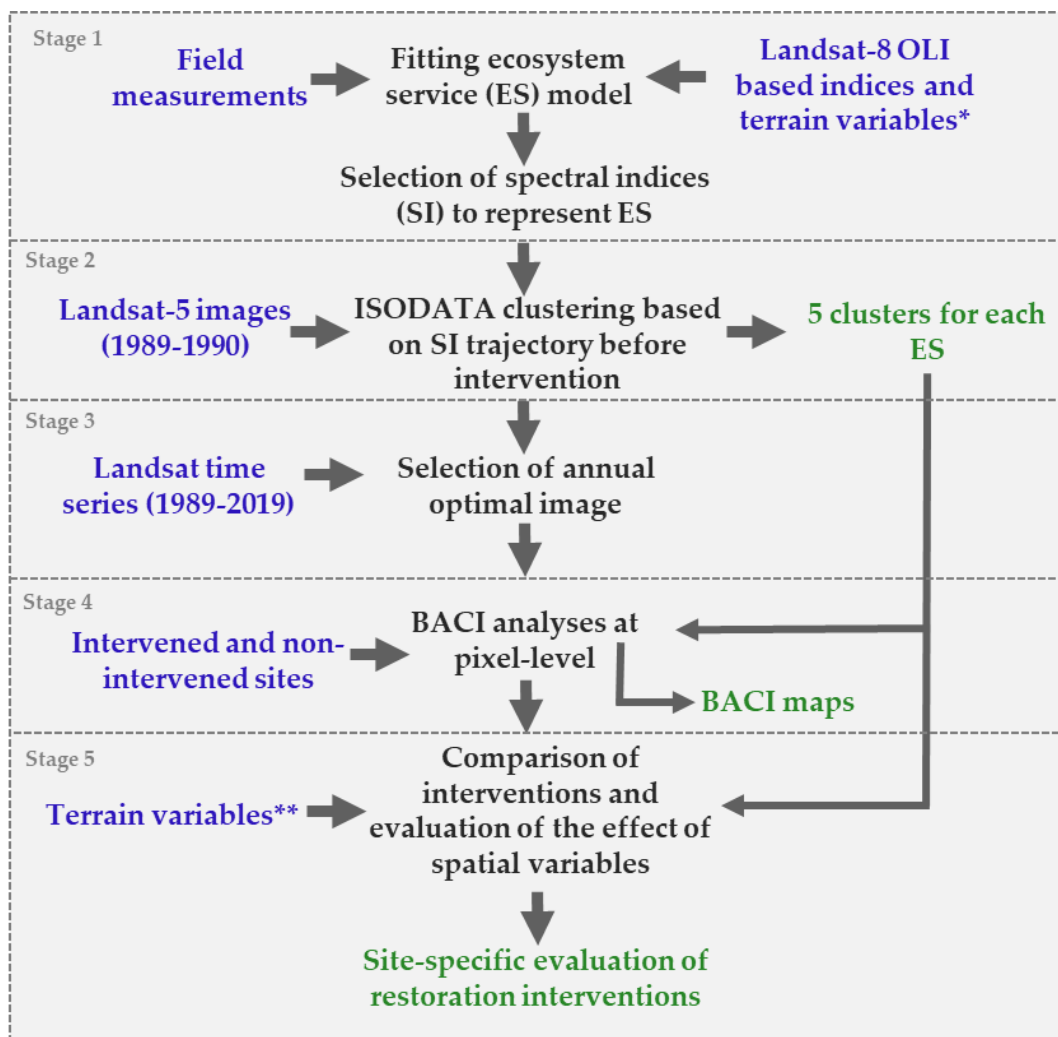

Fig 2.

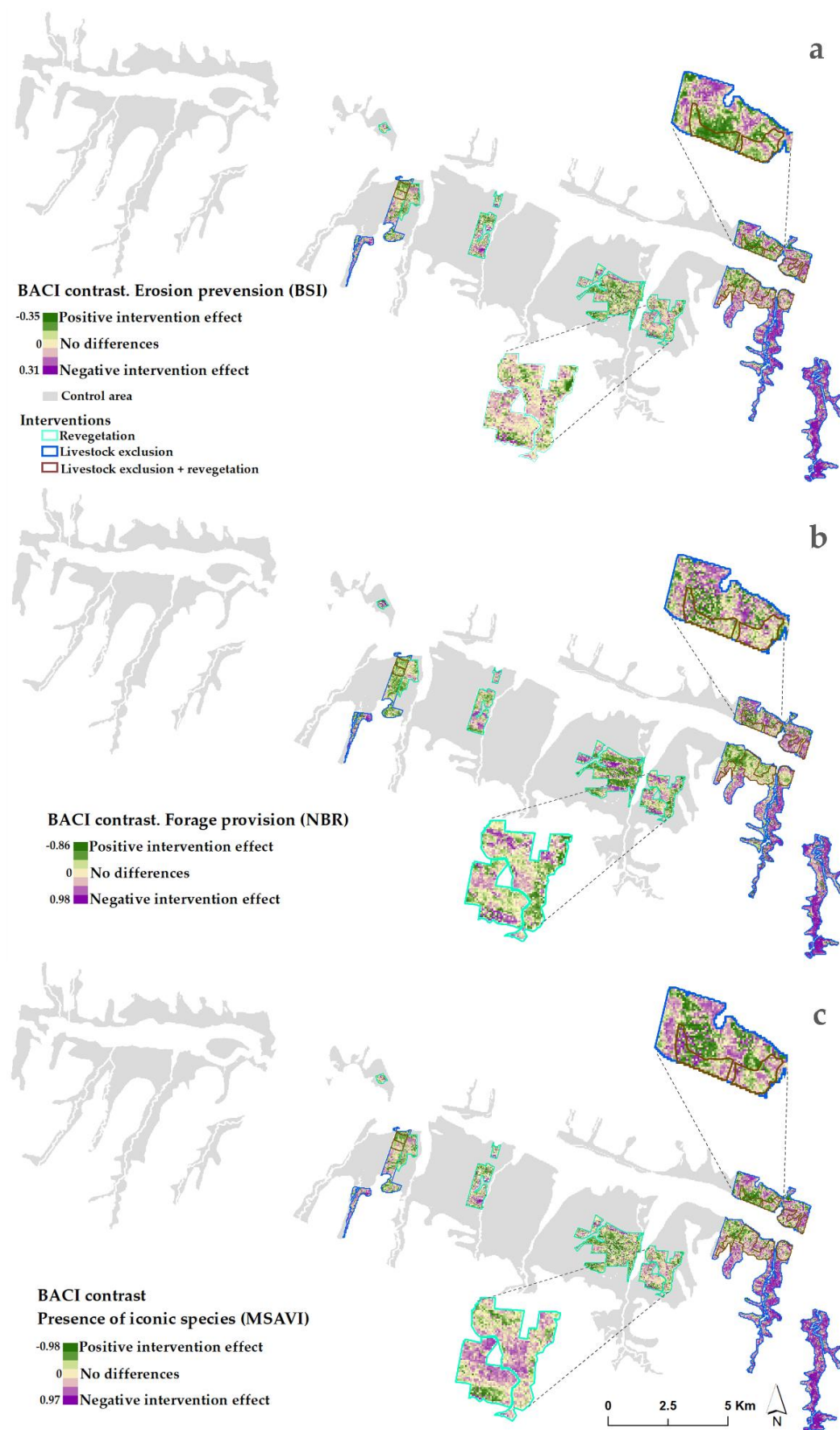

Fig 3.

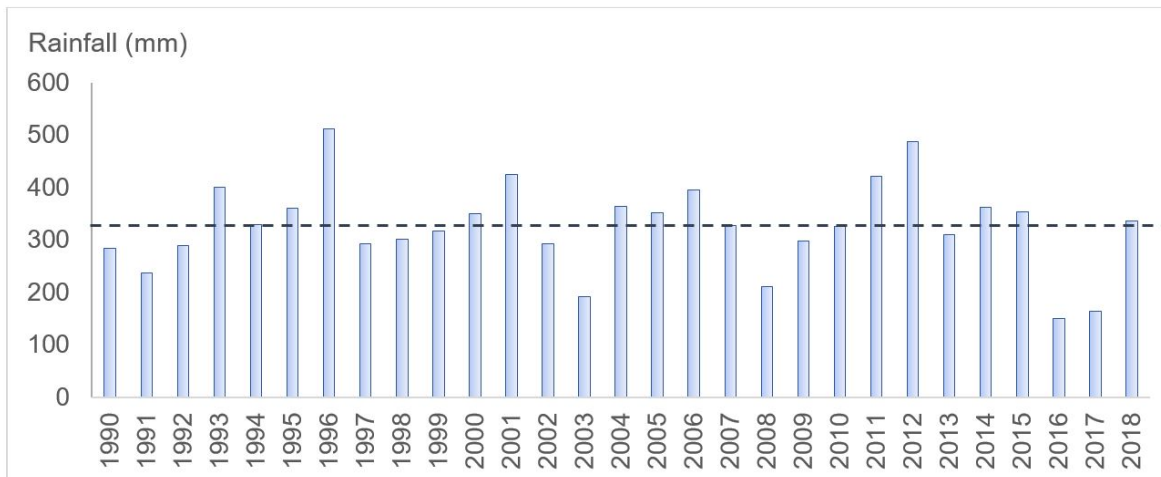

Fig S1.

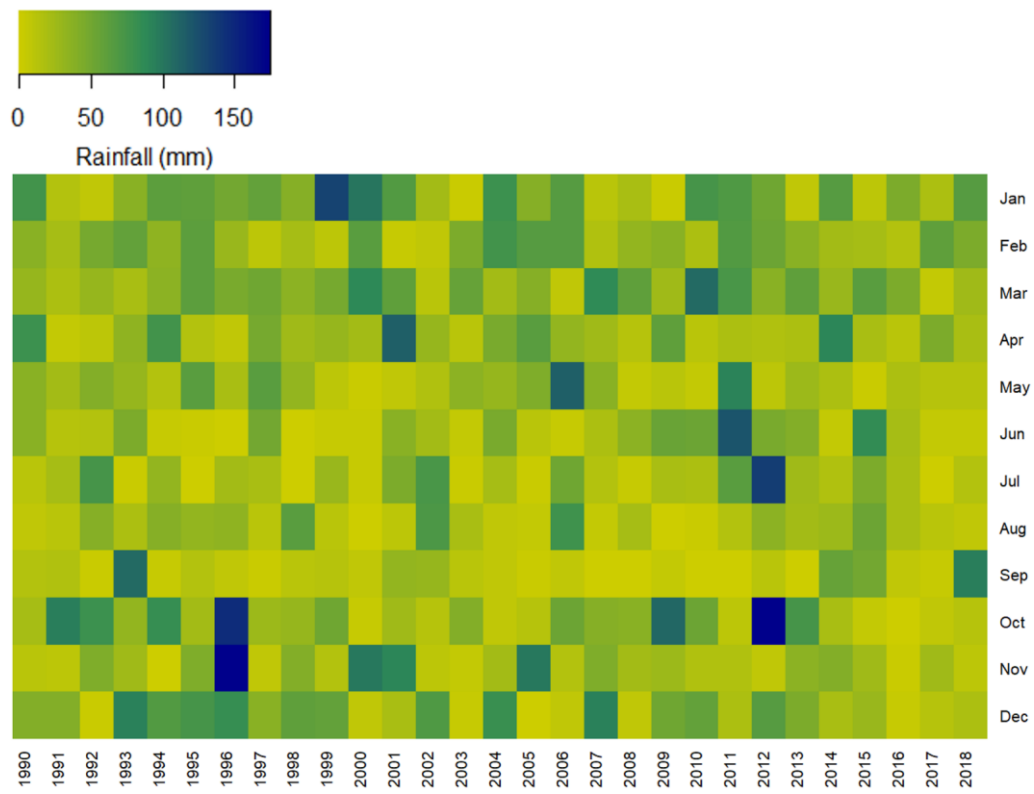

Fig S2.

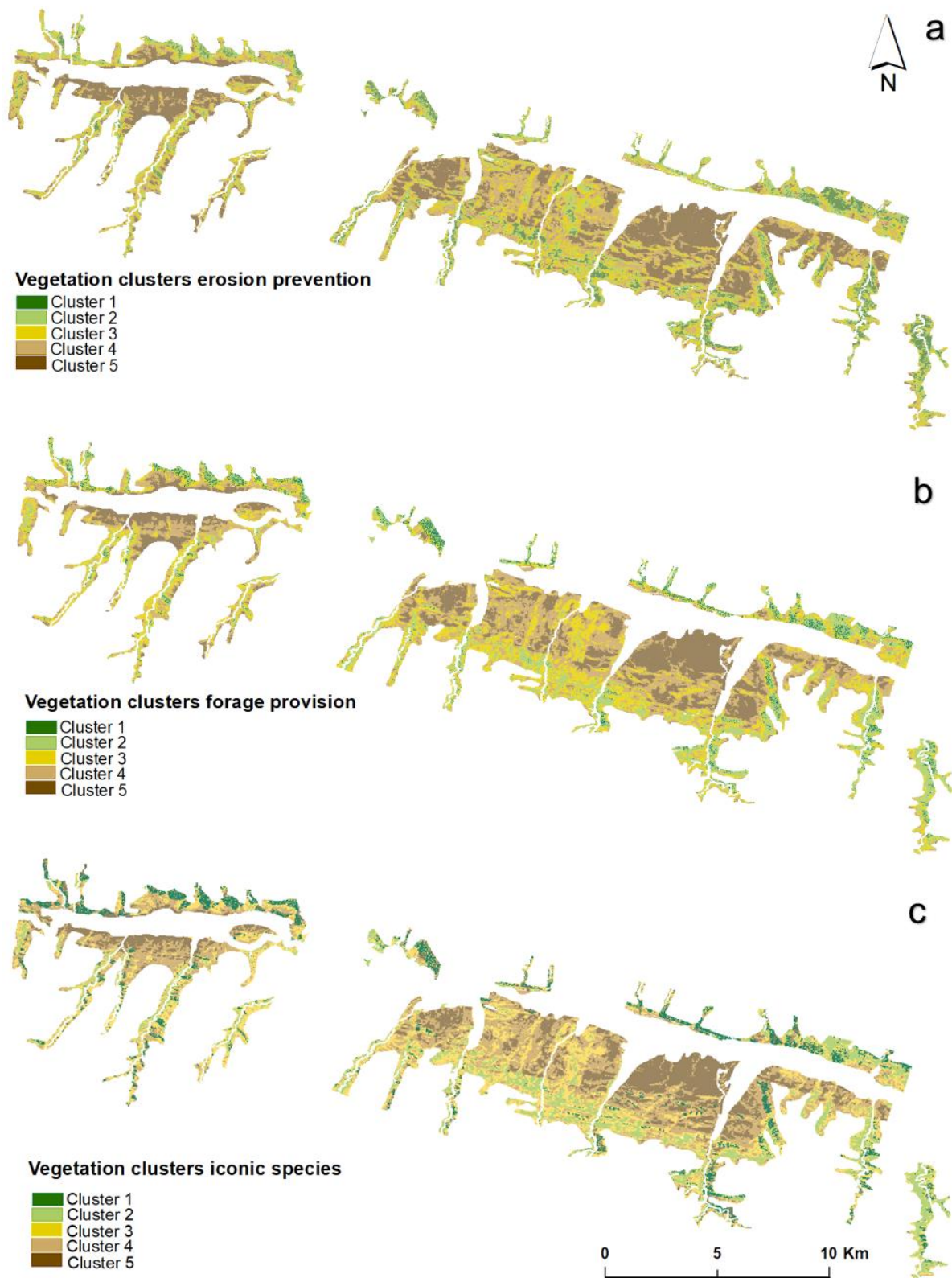

Fig S3.

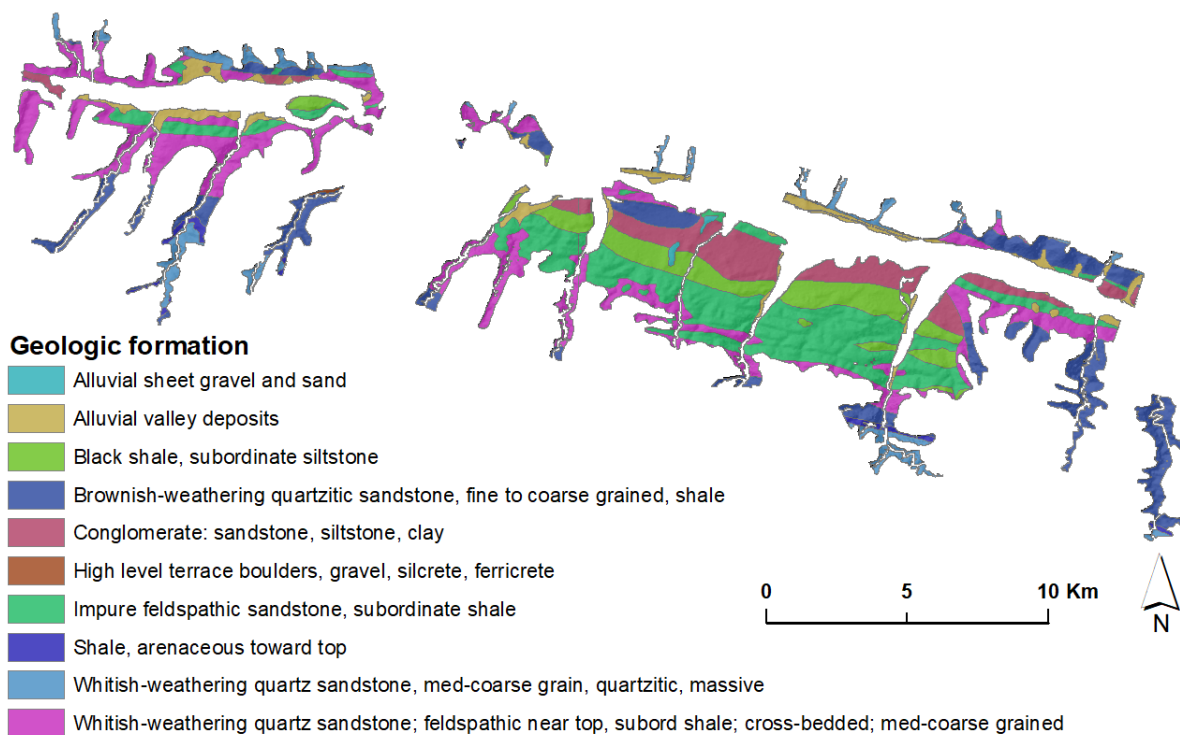

Fig S4.

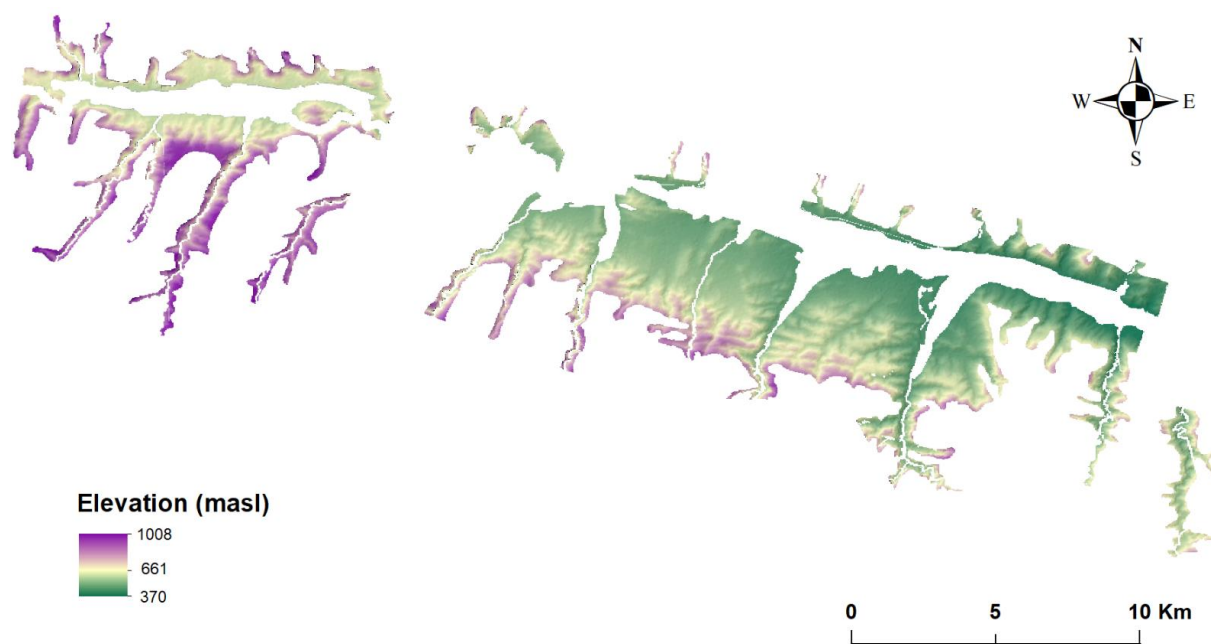

Fig S5.

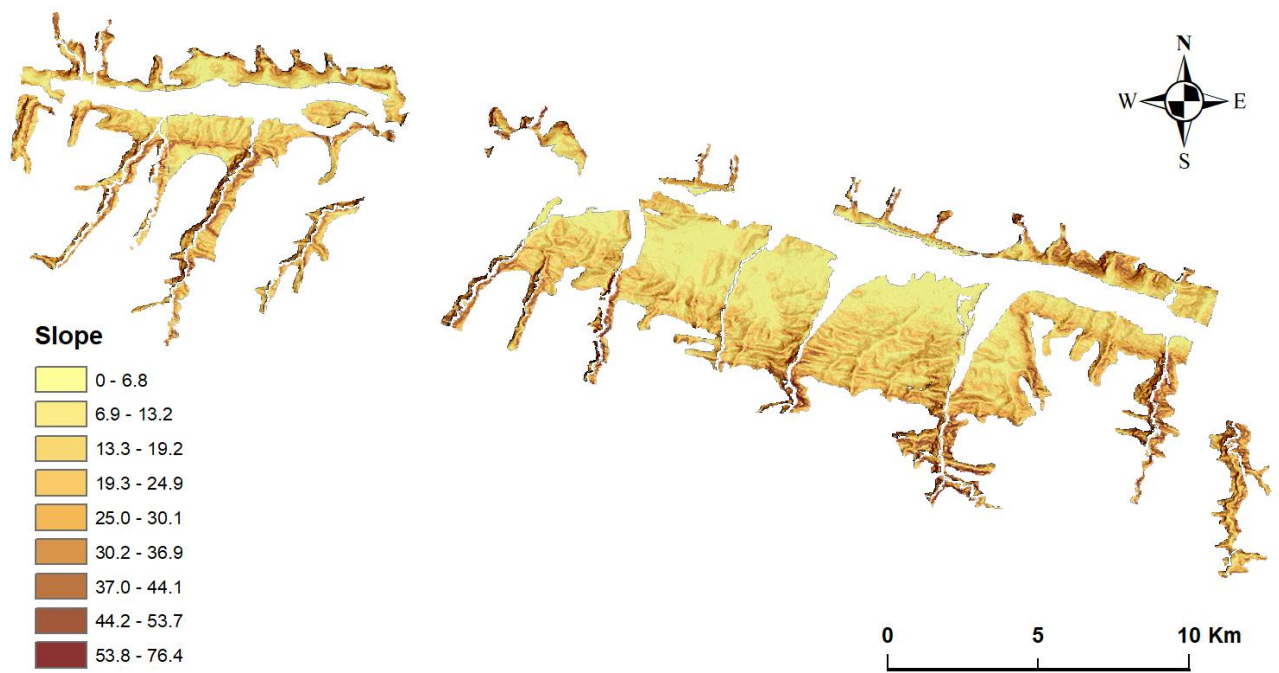

Fig S6.

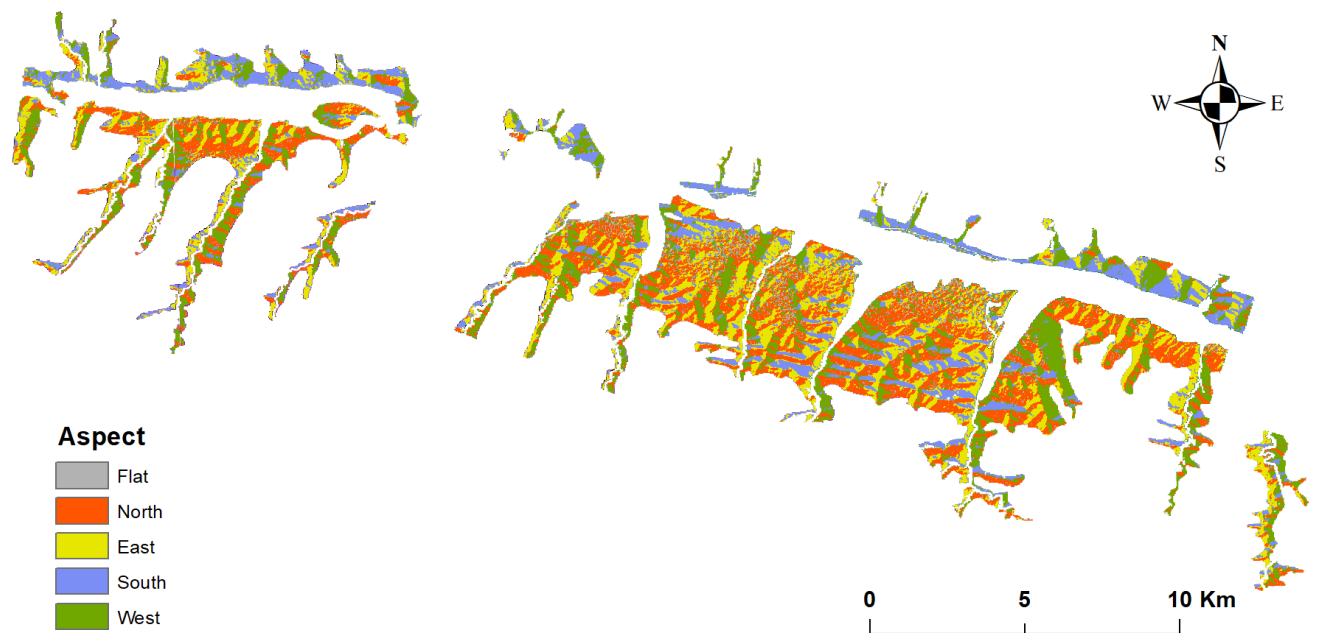

Fig S7.

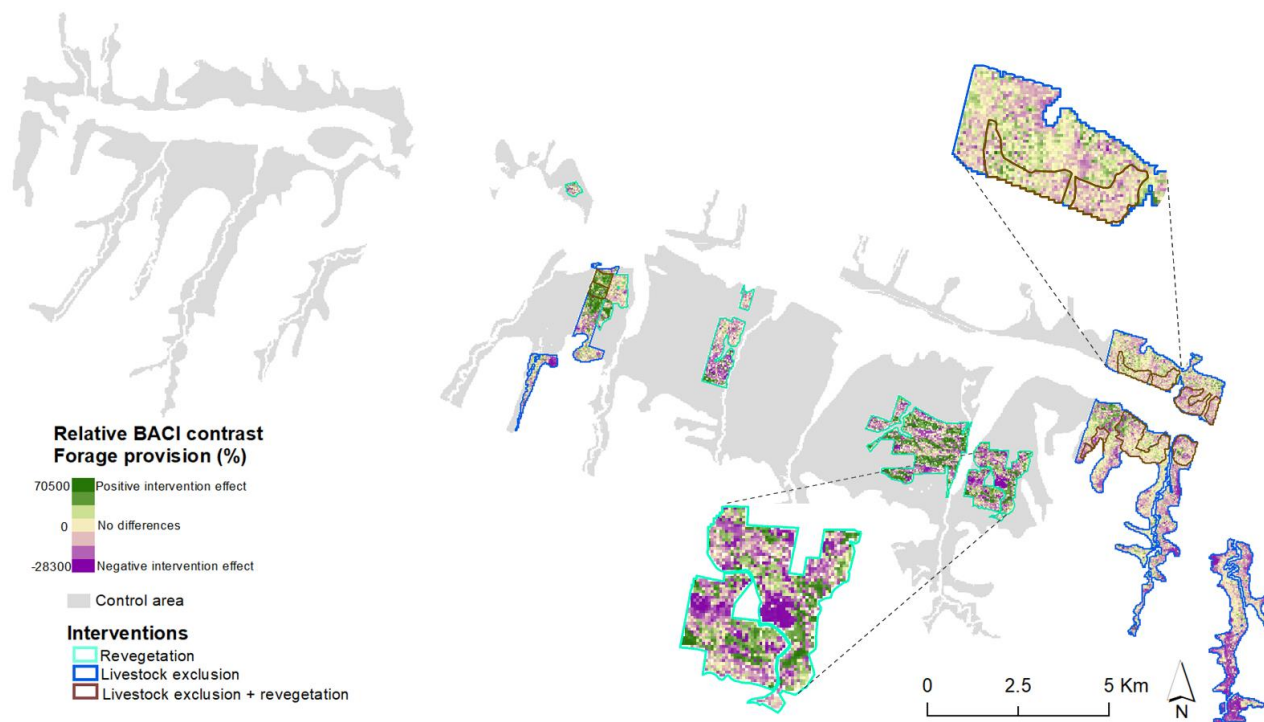

Fig S8.

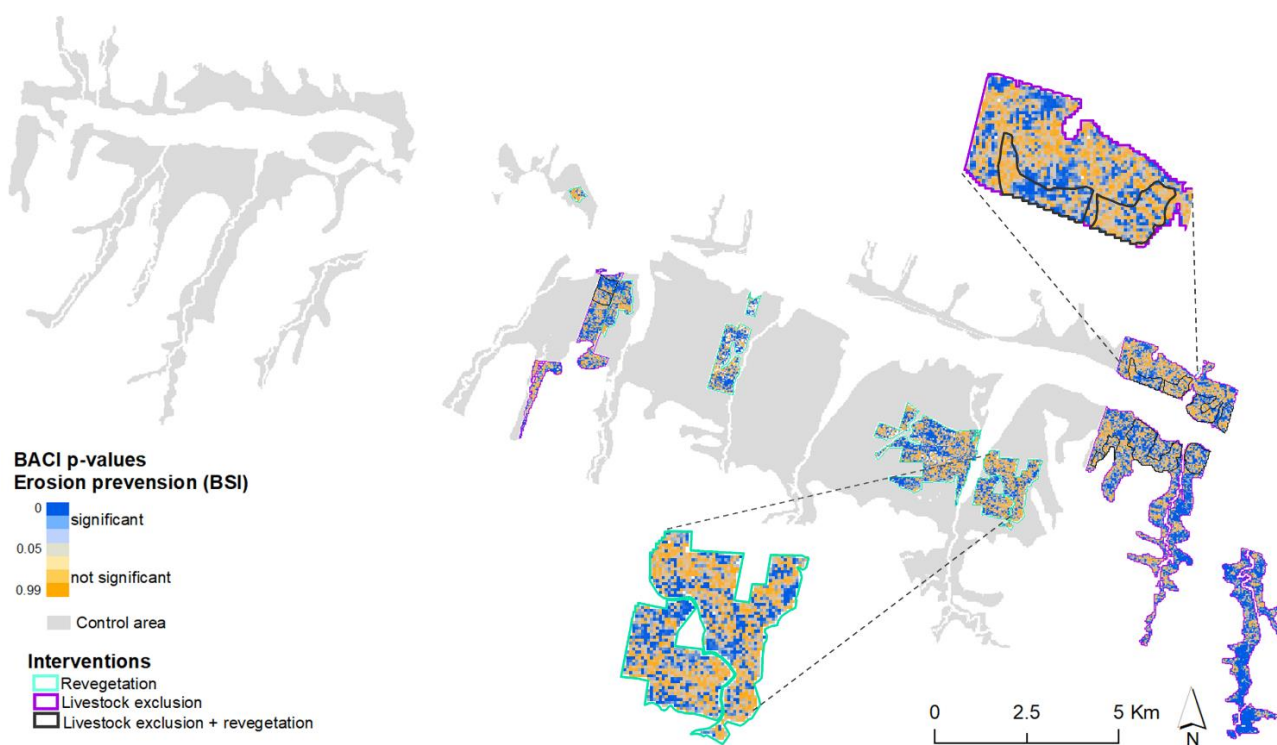

Fig S9.

Supplement: S1 Raw images — (PDF) [file pone.0243020.s002.pdf]
