# Supplementary material for: Long-term assessment of ecosystem services at ecological restoration sites using Landsat time series
Source: PLoS One. 2021 Jun 23;16(6):e0243020. doi: 10.1371/journal.pone.0243020 (PMC8221468; doi:10.1371/journal.pone.0243020)
Supplement: Attachment — Submitted filename: PONE-D-20-35781_reviewerComments.pdf [file pone.0243020.s003.pdf]

PLOS ONE

Long-term assessment of ecosystem services at ecological restoration sites using Landsat time series

--Manuscript Draft--

**Manuscript Number:**	PONE-D-20-35781
**Article Type:**	Research Article
**Full Title:**	Long-term assessment of ecosystem services at ecological restoration sites using Landsat time series
**Short Title:**	Long-term assessment of ecosystem services at ecological restoration sites using Landsat time series
**Corresponding Author:**	Trinidad del Río<br>University of Twente<br>Enschede, Overijssel NETHERLANDS
**Keywords:**	remote sensing; arid landscapes; revegetation; livestock exclusion; BACI
**Abstract:**	Reversing ecological degradation through restoration activities is a key societal challenge of the upcoming decade. However, lack of evidence on the effectiveness of restoration interventions leads to inconsistent, delayed, or poorly informed statements of success, hindering the wise allocation of resources, representing a missed opportunity to learn from previous experiences. This study contributes to a better understanding of spatial and temporal dynamics of ecosystem services at ecological restoration sites. We developed a method using Landsat satellite images combined with a Before-After-Control-Impact (BACI) design, and applied this to an arid rural landscape, the Baviaanskloof in South Africa. Since 1990, various restoration projects have been implemented to halt and reverse degradation. We applied the BACI approach at pixel-level comparing the conditions of each intervened pixel (impact) with 20 similar control pixels. By evaluating the conditions before and after the intervention, we assessed the effectiveness of long-term restoration interventions distinguishing their impact from environmental temporal changes. The BACI approach was implemented with Landsat images that cover a 30-year period at a spatial resolution of 30 m. We evaluated the impact of three interventions (revegetation, livestock exclusion, and the combination of both) on three ecosystem services; forage provision, erosion prevention, and presence of iconic vegetation. We also evaluated whether terrain characteristics could partially explain the variation in impact of interventions. The resulting maps showed spatial patterns of positive and negative effects of interventions on ecosystem services. Intervention effectiveness differed between land cover vegetation clusters, terrain aspect, and soil parent material. Our method allows for spatially explicit quantification of the long-term restoration impact on ecosystem service supply, and for the detailed visualization of impact across an area. This pixel-level analysis is specifically suited for heterogeneous landscapes, where restoration impact not only varies between but also within restoration sites.
**Order of Authors:**	Trinidad del Río<br>Louise Willemen<br>Anton Vrieling<br>Andy Snoeys<br>Andy Nelson
**Additional Information:**	
**Question**	**Response**
**Financial Disclosure**<br><br>Enter a financial disclosure statement that describes the sources of funding for the work included in this submission. Review	The authors received no specific funding for this work.

Powered by Editorial Manager® and ProduXion Manager® from Aries Systems Corporation

the submission guidelines for detailed requirements. View published research articles from *PLOS ONE* for specific examples.<br><br>This statement is required for submission and **will appear in the published article** if the submission is accepted. Please make sure it is accurate.<br><br>**Unfunded studies**<br>Enter: *The author(s) received no specific funding for this work.*<br><br>**Funded studies**<br>Enter a statement with the following details:<br>• Initials of the authors who received each award<br>• Grant numbers awarded to each author<br>• The full name of each funder<br>• URL of each funder website<br>• Did the sponsors or funders play any role in the study design, data collection and analysis, decision to publish, or preparation of the manuscript?<br>• **NO** - Include this sentence at the end of your statement: *The funders had no role in study design, data collection and analysis, decision to publish, or preparation of the manuscript.*<br>• **YES** - Specify the role(s) played.<br><br>* typeset	
**Competing Interests**<br><br>Use the instructions below to enter a competing interest statement for this submission. On behalf of all authors, disclose any competing interests that could be perceived to bias this work—acknowledging all financial support and any other relevant financial or non-financial competing interests.<br><br>This statement **will appear in the published article** if the submission is accepted. Please make sure it is accurate. View published research articles from *PLOS ONE* for specific examples.	The authors have declared that no competing interests exist

**NO authors have competing interests**<br><br>Enter: *The authors have declared that no competing interests exist.*<br><br>**Authors with competing interests**<br><br>Enter competing interest details beginning with this statement:<br><br>*I have read the journal's policy and the authors of this manuscript have the following competing interests: [insert competing interests here]*<br><br>* typeset	
**Ethics Statement**<br><br>Enter an ethics statement for this submission. This statement is required if the study involved:<br><br>• Human participants<br>• Human specimens or tissue<br>• Vertebrate animals or cephalopods<br>• Vertebrate embryos or tissues<br>• Field research<br><br>Write "N/A" if the submission does not require an ethics statement.<br><br>General guidance is provided below. Consult the submission guidelines for detailed instructions. **Make sure that all information entered here is included in the Methods section of the manuscript.**	N/A

Powered by Editorial Manager® and ProduXion Manager® from Aries Systems Corporation

**Format for specific study types**<br><br>**Human Subject Research (involving human participants and/or tissue)**<br>• Give the name of the institutional review board or ethics committee that approved the study<br>• Include the approval number and/or a statement indicating approval of this research<br>• Indicate the form of consent obtained (written/oral) or the reason that consent was not obtained (e.g. the data were analyzed anonymously)<br><br>**Animal Research (involving vertebrate animals, embryos or tissues)**<br>• Provide the name of the Institutional Animal Care and Use Committee (IACUC) or other relevant ethics board that reviewed the study protocol, and indicate whether they approved this research or granted a formal waiver of ethical approval<br>• Include an approval number if one was obtained<br>• If the study involved *non-human primates*, add *additional details* about animal welfare and steps taken to ameliorate suffering<br>• If anesthesia, euthanasia, or any kind of animal sacrifice is part of the study, include briefly which substances and/or methods were applied<br><br>**Field Research**<br><br>Include the following details if this study involves the collection of plant, animal, or other materials from a natural setting:<br>• Field permit number<br>• Name of the institution or relevant body that granted permission	
**Data Availability**<br><br>Authors are required to make all data underlying the findings described fully available, without restriction, and from the time of publication. PLOS allows rare exceptions to address legal and ethical concerns. See the PLOS Data Policy and FAQ for detailed information.	No - some restrictions will apply

A Data Availability Statement describing where the data can be found is required at submission. Your answers to this question constitute the Data Availability Statement and **will be published in the article**, if accepted.<br><br>**Important:** Stating 'data available on request from the author' is not sufficient. If your data are only available upon request, select 'No' for the first question and explain your exceptional situation in the text box.<br><br>Do the authors confirm that all data underlying the findings described in their manuscript are fully available without restriction?	
**Describe where the data may be found in full sentences. If you are copying our sample text, replace any instances of XXX with the appropriate details.**<br><br>• If the data are **held or will be held in a public repository**, include URLs, accession numbers or DOIs. If this information will only be available after acceptance, indicate this by ticking the box below. For example: *All XXX files are available from the XXX database (accession number(s) XXX, XXX.*).<br>• If the data are all contained **within the manuscript and/or Supporting Information files**, enter the following: *All relevant data are within the manuscript and its Supporting Information files.*<br>• If neither of these applies but you are able to provide **details of access elsewhere**, with or without limitations, please do so. For example:<br><br>*Data cannot be shared publicly because of [XXX]. Data are available from the XXX Institutional Data Access / Ethics Committee (contact via XXX) for researchers who meet the criteria for access to confidential data.*<br><br>*The data underlying the results presented in the study are available from (include the name of the third party*	All GIS files and codes will be available from the DANS Easy database

and contact information or URL).<br>• This text is appropriate if the data are owned by a third party and authors do not have permission to share the data.<br><br>* typeset	
Additional data availability information:	

Manuscript

Click here to access/download;Manuscript;BACImaps_Final.docx

Long-term assessment of ecosystem services at ecological restoration sites using Landsat time series

Trinidad del Río-Mena [1], Louise Willemen[1], Anton Vrieling[1], Andy Snoeys[2], Andy Nelson[1]

[1]Faculty of Geo-Information Science and Earth Observation (ITC), University of Twente, PO Box 217, 7500 AE Enschede the Netherlands; l.l.willemen@utwente.nl (LW); a.vrieling@utwente.nl (AV); a.nelson@utwente.nl (AN).

[2] Independent consultant; andy.snoeys@gmail.com

* Correspondence: t.delrio@utwente.nl; delriom.trini@gmail.com

Abstract:

Reversing ecological degradation through restoration activities is a key societal challenge of the upcoming decade. However, lack of evidence on the effectiveness of restoration interventions leads to inconsistent, delayed, or poorly informed statements of success, hindering the wise allocation of resources, representing a missed opportunity to learn from previous experiences. This study contributes to a better understanding of spatial and temporal dynamics of ecosystem services at ecological restoration sites. We developed a method using Landsat satellite images combined with a Before-After-Control-Impact (BACI) design, and applied this to an arid rural landscape, the Baviaanskloof in South Africa. Since 1990, various restoration projects have been implemented to halt and reverse degradation. We applied the BACI approach at pixel-level comparing the conditions of each intervened pixel (impact) with 20 similar control pixels. By evaluating the conditions before and after the intervention, we assessed the effectiveness of long-term restoration interventions distinguishing their impact from environmental temporal changes. The BACI approach was implemented with Landsat images that cover a 30-year period at a spatial resolution of 30 m. We evaluated the impact of three interventions (revegetation, livestock exclusion, and the combination of both) on three ecosystem services; forage provision, erosion prevention, and presence of iconic vegetation. We also evaluated whether terrain characteristics could partially explain the variation in impact of interventions. The resulting maps showed spatial patterns of positive and negative effects of interventions on ecosystem services. Intervention effectiveness differed between land cover vegetation clusters, terrain aspect, and soil parent material. Our method allows for spatially explicit quantification of the long-term restoration impact on ecosystem service supply, and for the detailed visualization of impact across an area. This pixel-level analysis is specifically suited for heterogeneous landscapes, where restoration impact not only varies between but also within restoration sites.

Keywords: remote sensing, arid landscapes, revegetation, livestock exclusion, BACI

1. Introduction

Rural landscapes depend on and simultaneously supply several ecosystem services, nature's contribution to people [1,2]. However, the quality of rural landscapes is deteriorating due to the expansion of croplands and grasslands into native vegetation and unsustainable agricultural practices [3]. Land degradation affects 40% of the agricultural land on earth, reducing the provision of ecosystem services and resulting in adverse environmental, social, and economic consequences [4–6]. It has been estimated that land degradation has a detrimental effect on 3.2 billion individuals and reflects an economic loss in the range of 10 percent of annual global gross product [3]. Given the increased pressure on ecosystems, restoration of degraded lands has become an important element of multiple global initiatives [7]. Several international initiatives have developed strategic targets as part of land sustainability agendas [e.g. 8–13] that are directly or indirectly linked to ecological restoration [14]. More recently, the United Nations (UN) declared 2021–2030 as the Decade on Ecological Restoration, with the aim of recognizing the need to accelerate global restoration of degraded ecosystems to mitigate negative impacts of climate change crisis protect biodiversity on the planet [15]. However, ineffective restoration efforts could inadvertently lead to a major waste of resources, continued deterioration of biodiversity and perceptions of conservation failure [16].

Restoration is defined as 'any intentional activity that initiates or accelerates the recovery of an ecosystem from a degraded state'; regardless of the form or intensity of degradation [17]. Restoration actions can vary from improving vegetation cover [e.g. 18,19] to diverse land management and policy implementations for improving the quality of terrestrial [e.g. 7,20,21], aerial [e.g. 22], or aquatic ecosystems [e.g. 23,24]. A successful ecological restoration should be effective, efficient and engaging through a collaboration of multiple stakeholders across sectors [25]. However, the basis of evidence to guide restoration practitioners is scarce, given the lack of long-term monitoring to determine the circumstances under which restoration efforts work [26]. This lack of impact evidence leads to incomplete, overdue or poorly informed claims of progress, hinders the effective allocation of resources

and represents a lost opportunity to select the best technologies and methods based on a critical evaluation of the lessons learned [27–29].

Monitoring and evaluating restoration interventions presents several challenges, including: i) restoration projects are often implemented across large areas with limited accessibility and large spatial heterogeneity; ii) the high economic costs and capacity constraints of field monitoring methodologies hinder the long-term documentation of restoration projects, particularly to assess the effects of such interventions outside their project timespan; iii) restoration initiatives often take a long time to start generating benefits [30]; iv) simple comparison of means between impact and control sites do not account for pre-existing differences between sites; v) after a restoration effort, ecosystem services show great variation in their temporal and spatial patterns and rate of change of the trajectories towards the desired reference [31]; and vi) and vi) observed state changes may also be attributable to intra- and inter-annual climate variability, making a direct comparison of conditions before and after insufficient [32].

Remote sensing (RS) plays an important role in studying complex interactions between natural and social systems [33], such as land management. RS provides a range of data with varying spatial and temporal extents, and resolutions, facilitating monitoring and mapping of the environment [34–36]. Time series derived from satellite data can identify both rapid and longer-term changes in vegetation cover [36,37]. Spectral information from optical satellite images allows for the quantitative estimation of biophysical vegetation characteristics, such as vegetation cover [e.g. 40], aboveground biomass [e.g. 41,42], leaf area, and leaf chlorophyll concentration [e.g. 41] among others. This remotely sensed information can be used to quantify, map, and monitor provisioning, regulating, and (to a lesser extent) cultural ecosystem services [42]. With over 30 years of directly comparable satellite observations, freely available Landsat imagery with moderate frequency (16 days) and medium resolution (30 m) can assess long-term dynamics of ecosystems [43,44] and allow for temporal comparisons of restoration sites [32].

Despite widespread awareness of the potential of RS, most ecosystem service studies use static land use/land cover information rather than a more dynamic assessment of satellite records [35,37,45,46]. However, land use/land cover maps are not always available [47], can lead to

generalization errors as they exclude spatial variation within the same vegetation category [35,48], and may be outdated or available only at large temporal intervals. In addition, most studies fail to take advantage of the long temporal records of available remotely sensed data, one of their great strengths in assessing ecosystem services [33,35]. Previous studies have shown that directly linking *in situ* observations of ecosystem services to remotely sensed data improves the capturing of their spatio-temporal dynamics as compared to the often-used practice of linking the service supply directly to one land cover class [49,50].

A method for assessing the impacts of natural or human-induced disturbances on ecosystems where the allocation of treatment and control sites cannot be randomized, is the before-after-control-impact/treatment (BACI) approach [51]. Among other applications, the BACI approach can be used to assess the impacts of long-term restoration interventions independently of natural temporal changes [52]. It compares the conditions of a restored area (impact) with the conditions of nearby unrestored (control) areas before and after the restoration intervention [53,54]. The BACI approach was recently applied using RS images to assess land restoration interventions in semi-arid landscapes in West-Africa [32] using 20 automatically selected control sites for each impact site and multiple years for their "before" and "after" periods. The use of several controls invalidates claims that the findings of the BACI assessment are primarily due to a weak choice of control sites [55]. In the respective study for West Africa [33], topographic variations were not explicitly accounted for, and intervention effectiveness was assessed for the entire impact site without considering terrain variation and within-site differences in interventions' effectiveness. These can however be important because different vegetation types grow in locations with different elevation, slope, aspect, and parent material (geological material from which soils are formed) [56–60]. The freely accessible collection of historical Landsat imagery can mitigate the widespread lack of timely, long-term, reliable, and homogeneous ground information for monitoring restoration interventions.

This study contributes to a better understanding of the spatial and long-term distribution of ecosystem service supply for supporting the site-specific evaluation of restoration interventions by

expanding the spatial scope of the BACI analyses to pixel-level. By analyzing intervention impact at 30 m pixel-level rather than for large intervention areas, we aim to capture variation and patterns of intervention outcomes within a heterogeneous landscape. The specific aims of this research are to: (1) quantify the effect of restoration interventions on ecosystem service supply using Landsat time series data and the BACI approach at pixel-level, and (2) evaluate whether terrain characteristics affect the spatial distribution of restoration effectiveness, using an arid agricultural landscape study area in South Africa as a case study.

2. Materials and methods

2.1. Study area and interventions

The study area is composed of the subtropical arid thickets and shrublands that are in the central and eastern area of the Baviaanskloof Hartland Bawarea Conservancy, Eastern Cape in South Africa (Figure 1). The considered intervened and control areas cover about 100 km^2. This hilly region consists of a mixture of large, private farms (between 500 and 7,600 hectares in size) and rural communal land. The mean annual rainfall is 327 mm over the last 30 years, with an erratic distribution across and within years [61] (Figure S1 and S2). The average annual temperature in the area is 17 °C. Temperatures of up to 40 °C are frequently reported for December to February, whereas temperatures between June and August may occasionally fall below 0 °C [62].

Dense thicket vegetation in the study area is dominated by spekboom (*Portulacaria afra*), which is grazed by small livestock and wildlife [63]. Most of the thicket has been heavily degraded by unsustainable pastoralism [64,65]. Because spekboom is a succulent species that propagates vegetatively [66], spontaneous recovery does not occur in heavily degraded sites [67,68]. Land degradation has resulted in severe and widespread soil erosion [62]. The reduction of the natural vegetation, which is the common source of food for the extensively farmed goat and sheep in the area, has also contributed to a dramatic decline in agricultural returns in recent years [69] and degradation of the aesthetic appeal of the landscape [70]. For more than a decade, the planting of spekboom cuttings has been practiced as

a practical method of restoration in the area [71–74]. Several farmers in the study area are transitioning from extensive goat and sheep farming to more sustainable farming activities such as essential oil production and agrotourism. Essential oil production is considered a more sustainable farming practice in the area as it requires limited water and fertilizer inputs and needs less land to be profitable, compared to goat farming. This transition is made in partnership with Commonland, Grounded, and Living Lands, which are three local and international non-governmental organizations. These organizations support large-scale and long-term restoration and sustainable land use initiatives.

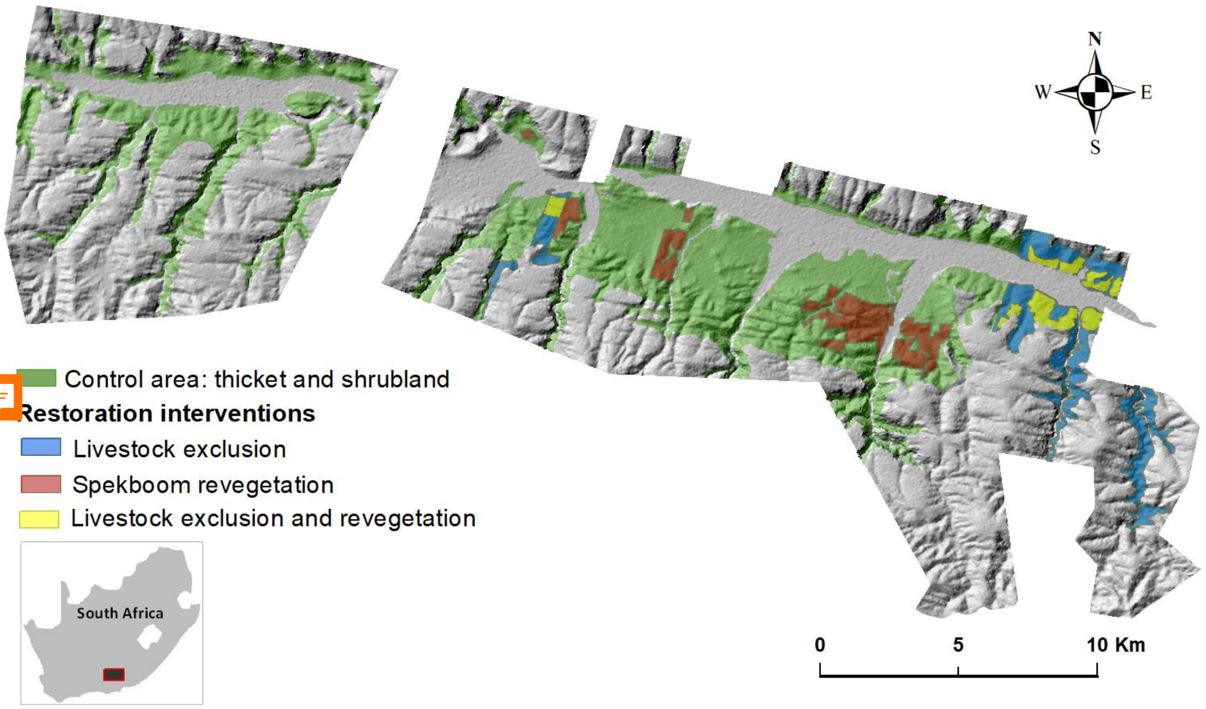

[illegible]igure 1. Restoration intervention sites in the in the Baviaanskloof Hartland Bawarea Conservancy study area in South Africa. Shading indicates topographic relief.

We assessed three restoration interventions:

1. **Spekboom revegetation**: Between 2010 and 2015, about 1,100 ha were planted with spekboom to reduce degradation trends and assist the recovery of the degraded thicket vegetation [72]. The planting of spekboom truncheons was implemented through the national Department of Environmental Affairs, Natural Resource Management directorate, Expanded Public Works Program (EPWP).

2. **Livestock exclusion**: This intervention covers approximately 7,400 ha of farmland where livestock has been removed from 1990 onwards to allow for natural revegetation that could potentially prevent erosion and provide forage among other ecosystem services.

3. **Combination of livestock exclusion and revegetation:** Over time, spekboom was planted in some of the livestock exclusion areas (337 ha approximately). We considered the combination of these two ecological restoration measures as a separate intervention.

Each of the restoration interventions aimed to address local environmental challenges associated with land degradation by improving ecosystem service supply. To illustrate, for this paper we selected three ecosystem services; one provisioning, one regulating, and one cultural (Table 1).

Table 1. Selected ecosystem services, their indicators, and their related intervention. Str.VC stands for stratified vegetation cover.

Ecosystem service	Ecosystem service indicator
Erosion prevention	Stratified vegetation cover index (% Str.VC) [75]
Forage provision	Green biomass (kg m^{-2}) [76]
Presence iconic species	Spekboom cover (%) [77]

2.2. General workflow

Our workflow is summarized in Figure 2. The first stage consisted of a) building ecosystem service models based on field measurements, remotely sensed spectral indices derived from Landsat 8 Operational Land Imager (OLI) and terrain variables (slope and elevation), and b) selecting the spectral index from the best-fit model as a proxy for each ecosystem service. As the terrain variables are assumed to remain constant over time, we only used the selected spectral indices to represent changes in ecosystem service supply. In the second stage, for each ecosystem service considered, we divided the landscape into five vegetation clusters based on the similarity in the trajectory of the spectral index that represents that ecosystem service. This was achieved by applying the ISODATA unsupervised classification for the Landsat time series acquired before any intervention was implemented. In the third stage, we selected one satellite image per analyzed year based on the highest vegetation indices and lower bare soil index separately for each ecosystem service. In the fourth stage, we estimated the

intervention impact for each pixel using BACI contrast for each ecosystem service at the restoration sites. Finally, the resulting BACI contrast values were analyzed to spatially evaluate the intervention impacts, and to assess if this impact was different for vegetation clusters, terrain aspect classes, and soil parent material.

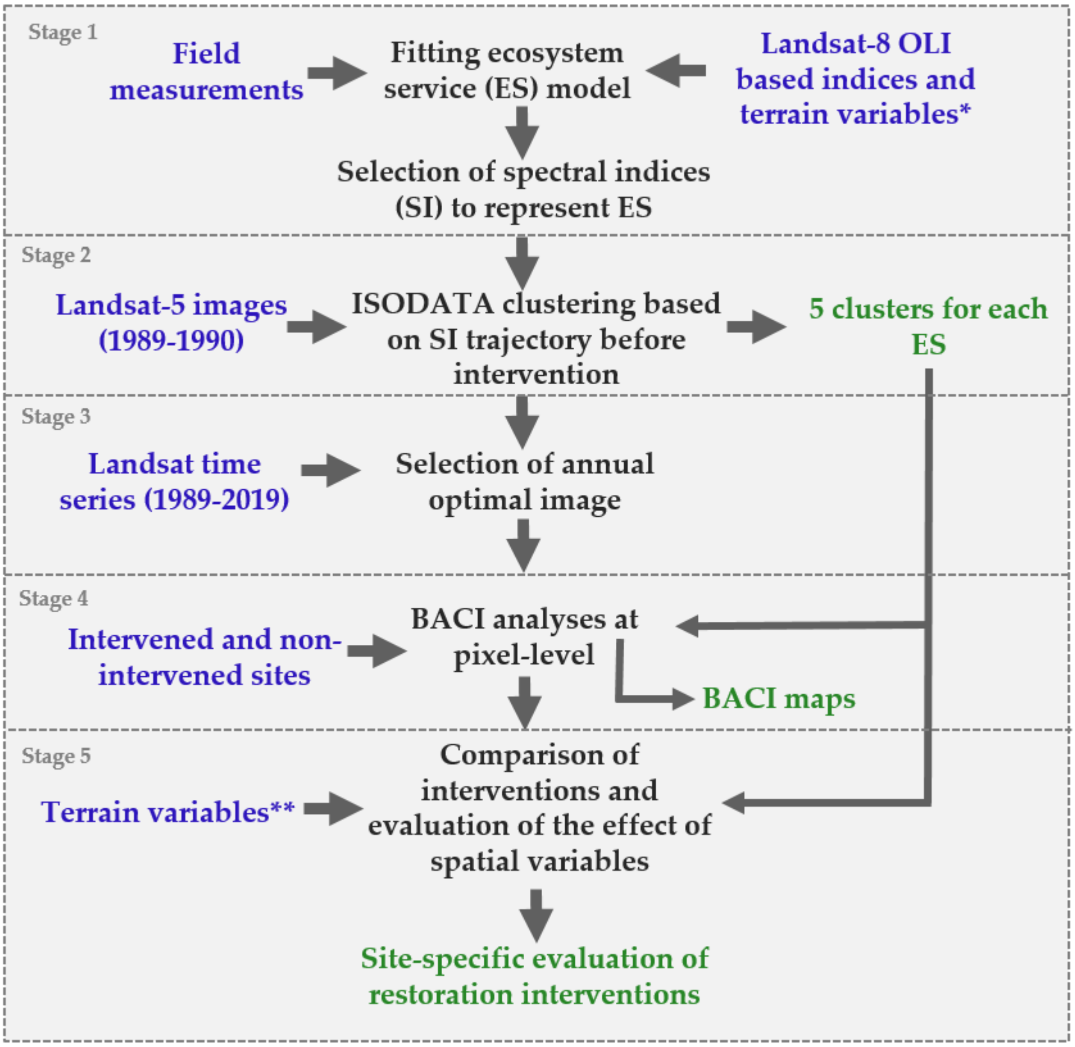

Figure 2. Workflow diagram. The methodological steps are referred to as stages (in grey). Blue colors indicate input data; black shows processes and analyses; green corresponds to output data.
* In stage 1 we used slope and altitude.
** In stage 5 we used slope and altitude in the correlation analyses, and aspect and parent material for the nonparametric test.

2.3. Model calibration and selection of spectral indices to represent ecosystem services

During the fieldwork period from May and July 2017, we estimated ecosystem services based on measurements in 32 plots of 900 m² that were distributed over the study area [77]. Field measurements included canopy dimensions and vegetation cover. We calculated stratified vegetation cover to quantify erosion prevention [75] by combining the field-measured fractional vegetation cover of different

vegetation types. The presence of iconic species was estimated from field-based estimations of spekboom cover. For the forage provision we used previously developed allometric equation to estimate green biomass based on measured vegetation cover for grasses and shrubs [76].

We fitted linear and non-linear regression models using ten spectral indices (Table 2) derived from Landsat 8 OLI acquired on 14/05/2017 and terrain variables to identify a relationship between RS and field-based estimates of the ecosystem services. To avoid multi-collinearity between predictor variables, we only considered models having a variance inflation factor (VIF) lower than 5.0 [78] using the R caret package [79]. For each ecosystem service, we selected the most representative Landsat spectral index based on the best performing model according to the lowest Akaike Information Criterion (AIC) [80], using the multi-model inference (MuMIn) R package [81]. We used a five-fold cross-validation to test the models, which we repeated 100 times [82] using the 'caret' R package [79]. We used a cross-validation approach, as our limited sample size did not allow us to hold back data for independent model validation. Finally, we used another Landsat 8 OLI image matching the fieldwork period (17/07/2017) to check for consistency of the prediction of the fitted ecosystem service model at a different moment.

Table 2. Spectral indices and their equations used to explain ecosystem services measured in the field. NIR = Near infrared; SWIR = short wave infrared; the SWIR 1 band measures radiation in the 1.57-1.65 μm wavelength domain and SWIR 2 in 2.11-2.29 μm; G (gain factor) = 2.5; Coefficients L=1, C1 = 6, C2 = 7.5

Index	Index equation
Normalized Difference Vegetation Index (NDVI)	(NIR - Red) / (NIR + Red)
Soil Adjusted Vegetation Index (SAVI)	((NIR - Red) / (NIR + Red + L)) * (1 + L)
Soil Adjusted Vegetation Index (MSAVI)	(2 * NIR + 1 – sqrt ((2 * NIR + 1)^2 – 8 * (NIR - R))) / 2
Enhanced Vegetation Index (EVI)	EVI = G* ((NIR - Red) / (NIR + C1 * Red – C2 * Blue + L))
Normalized Pigment Chlorophyll Ratio Index (NPCRI)	(Red - Blue) / (Red + Blue)
Bare Soil Index (BSI)	(SWIR1 + Red) – (NIR + Blue) / (SWIR1 + Red) + (NIR + Blue)
*Normalized Burned Ratio (NBR)	(NIR – SWIR2) / (NIR + SWIR2)
Normalized Burned Ratio 2 (NBR2)	(SWIR1 – SWIR2) / (SWIR1 + SWIR2)
Normalized Difference Moisture Index (NDMI)	(NIR - SWIR1) / (NIR + SWIR1)
Normalized Difference Water Index (NDWI)	(Green - NIR) / (Green + NIR)

*Note that NBR is sometimes named differently and is not only used for detecting burned areas, i.e. Infra-Red Index, Normalized Difference Infrared Index and Shortwave Vegetation Index [83].

2.4. RS- GIS data description

The RS and other spatial data used in this study are summarized in Table 3 and organized according to their respective methodological stage (Figure 2). All used images from Landsat 5, 7, and 8 were from path 172 and row 83, and accessed through Google Earth Engine (GEE). We used the Landsat Level-2 Surface Reflectance Science Product, courtesy of the U.S. Geological Survey [84], which are derived from the Landsat Collection 1 Tier 1 dataset. All selected images were cloud-free in the restoration sites (Figure 1) and small clouds were masked out in control areas. Scenes having more than 5% clouds in the control areas were excluded. For each retained image, we then extracted the relevant spectral indices. The dates of the selected images are listed on Tables S1 and S2. In addition to the RS data, we used elevation (meters above sea level), slope (degrees), aspect and soil parent material maps (Figures S3-6).

Table 3. Spatial input data for methodological stages.

Methodological Stage	Variables	Description	Data source
Stage 1	10 spectral indices	Landsat- 8 OLI from 14/05 and 17/07, 2017	USGS [84]
	Slope and elevation	12.5 m resolution DEM derived from ALOS PALSAR	Geophysical Institute of the University of Alaska Fairbanks [85]
Stage 2	Time series of spectral indices	Extracted from Landsat 5 TM (26/02/1989 to 27/10/1990)	USGS [84]
Stage 3	Spectral index values	Landsat 5, 7 and 8 images. Period 'before': 1989, 1990; 2007 to 2014 (depending on intervention). Period 'after': 2017-2019	USGS [84]
Stage 4	Restoration sites	Type and lifespan	Provided by Living Lands
Stage 5	Slope, aspect, and elevation	12.5 m resolution DEM derived from ALOS PALSAR	Geophysical Institute of the University of Alaska Fairbanks [78]
	Parent material	In the study area: black shale, shale, Enon conglomerate, feldspathic sandstone, quarzitic sandstone, alluvium, and terrace gravel	South African Council for Geoscience [86]

2.5. ISODATA clustering, BACI analyses, and intervention evaluation

To locate areas having similar vegetation characteristics within thicket vegetation before any of the assessed interventions started, we performed an ISODATA clustering using the multi-temporal

trajectory of the selected index for each ecosystem service [87]. The clustering was performed in ERDAS IMAGINE using the ten available cloud free Landsat 5 Thematic Mapper (TM) images from 26/02/1989 to 27/10/1990 (Table S1) based on how similar the trajectory of the spectral index was between pixels. We used up to 50 iterations and a convergence limit of 1. We arbitrarily limited the prior vegetation characteristics to five vegetation clusters with the intention of distinguishing the key groups with varying temporal behavior before the interventions took place, following the procedure described in [32].

To calculate the BACI contrast for the approximately 22600 intervened pixels we selected Landsat images representing the greenest moment of the year. This moment is defined by calculating the average highest vegetation index -or lowest bare soil index value- for the study area (i.e. maximum MSAVI or minimum BSI value) (Table S2). Pixels falling within the Landsat 7 Enhanced Thematic Mapper Plus (ETM+) Scan Line Corrector (SLC) off data were excluded. We considered three years for the period before and after intervention, with exception of the interventions 'livestock exclusion' and 'livestock exclusion + revegetation' for which not enough images were available before 1989 and consequently we used only two years for the period 'before'. To focus the comparison on sites with a similar reference state, the BACI analyzes was carried out separately for each cluster. Secondly, for each of the intervened pixels, we obtained the spectral index values for each of the assessed years. We randomly selected 20 control pixels per intervened pixel [32]. We developed a simple Windows command line application to randomly select the control pixels from the same vegetation clusters as the intervened site, avoiding pixels within the SLC off data from Landsat 7 ETM+. Using the same command line application, we extracted the spectral index values for each intervened pixel and its respective control pixels for each year of the period before and after the intervention. We then calculated the BACI contrast based on the following formula:

$$\text{BACI contrast} = (\mu_{CA} - \mu_{CB}) - (\mu_{IA} - \mu_{IB})$$

where μ is the temporal (selected years) and spatial (20 controls) mean of the variables selected to represent the impact (in this study the selected vegetation indices); the letters C and I stand for control

and impact, respectively; and the letters B and A stand for the periods "before and "after", respectively. By convention, a negative contrast indicates that the variable (except the BSI index, which is a proxy for percentage bare soil instead of vegetation) has increased more (or decreased less for BSI) in the impact site with respect to the control sites during the time period ranging from before to after the implementation of the restoration project. The BACI contrast is expressed in the same units of the variable of interest, i.e. the spectral index used, and consequently is unitless in our case.

Since our data did not pass the Shapiro–Wilk test for normality, we used a nonparametric Kruskal-Wallis test (Games-Howell post-hoc test at 0.05) to explore the differences between restoration interventions, vegetation clusters, terrain aspect, and soil parent material on the BACI contrast. We randomly sampled pixels for each compared group (i.e. intervention, cluster number, aspect or parent material, applying a minimum distance of 60 meters between points to avoid selecting neighboring pixels and ensure independent samples. We selected the five parent materials classes that represent the largest areas in intervened sites (Table 4). We also checked for association between the BACI contrast of each restoration intervention and ecosystem service with slope and elevation by fitting regression models, using random samples of 10% of the data.

Table 4. Selected parent material classes for BACI contrast comparison representing the largest areas of intervened sites.

Parent materials classes	Description
Quartzitic sandstone (shale) (Sg)	Brownish weathering quartzitic sandstone, fine to coarse grained, shale
Feldspathic sandstone (S-Db)	Impure feldspathic sandstone, subordinate shale
Shale (Dg)	Black shale, subordinate siltstone
Enon conglomerate (Je)	Conglomerate: sandstone, siltstone, clay
Quartzitic sandstone (Ss)	Whitish-weathering quartz sandstone; feldspathic near top, subordinate shale; cross-bedded; med-coarse grained

3. Results

3.1. Selection of spectral indices

The RS-based models that best describe ecosystem services as measured in the field are presented in Table 5. The best model for erosion prevention is a second-degree polynomial fit with the BSI index. Forage provision was also fitted to second-degree polynomials using the NBR. The presence of iconic

species is the only ecosystem service described with a linear regression model that uses two predicting variables (MSAVI and elevation), where elevation contributed by 22% to the model (expressed as partial R^2). The R^2 in Table 5 represents the mean R^2 obtained from the repeated cross-validation. We used another image matching the fieldwork period to check if the models were consistent for different dates. The models based on spectral information for 17/07/2017 resulted in lower R^2 as compared to when spectral information for the validation data14/05/2017 was used for erosion prevention and forage provision. The R^2 of presence of iconic species increased by 2% for the validation date.

Table 5. Selected ecosystem service models based on indices derived from calibration between field measurements with Landsat 8 (14/05/2017) data and terrain variables. StrVC = Stratified vegetation cover, GB = Green biomass, SbC = Spekboom cover.

**Ecosystem service**	**Function**	**R^2**	**Standardized RMSE**	**n**	**R^2 validation date (17/07/2017)**
Erosion prevention	StrVC = 56.36(BSI–^{2} - 36.66(BSI) + 6.06	0.85	0.19	32	0.71
Forage provision	GB = 47.62(NBR)^2 + 55.55(NBR) + 8.71	0.71	1.19	32	0.67
Presence of iconic species	SbC = 56.36(MSAVI) + 18.05(Elevation) - 16.93	0.53	0.62	20	0.54

3.2. RS-based BACI analyses

The maps showing the BACI contrast of the assessed ecosystem services of approximately 22600 intervened pixels at 30 m resolution are presented in Figure 3. All the assessed ecosystem services show similar general patterns of positive and negative effects of interventions. However, when zooming in, at pixel level their BACI contrasts show differences. For correct interpretation of the results for erosion prevention, it is important to consider that the BSI index behaves inversely as compared to the other vegetation indices as it shows bare soil cover. Therefore, negative BACI contrast values for BSI indicate a positive effect on erosion control of the intervention. In this section, we use the term "better" to indicate higher NBR and MSAVI and lower BSI values, or their derived BACI contrasts. Even though in most areas the BACI contrast presents similar patterns for the three ecosystem services, the magnified areas in Figure 3-5 also show areas with clear differences in BACI contrast for the different ecosystem services. In addition, the maps show high spatial variability of restoration interventions impact within restoration sites. Within a single intervention, ecosystem service supply both increased and decreased.

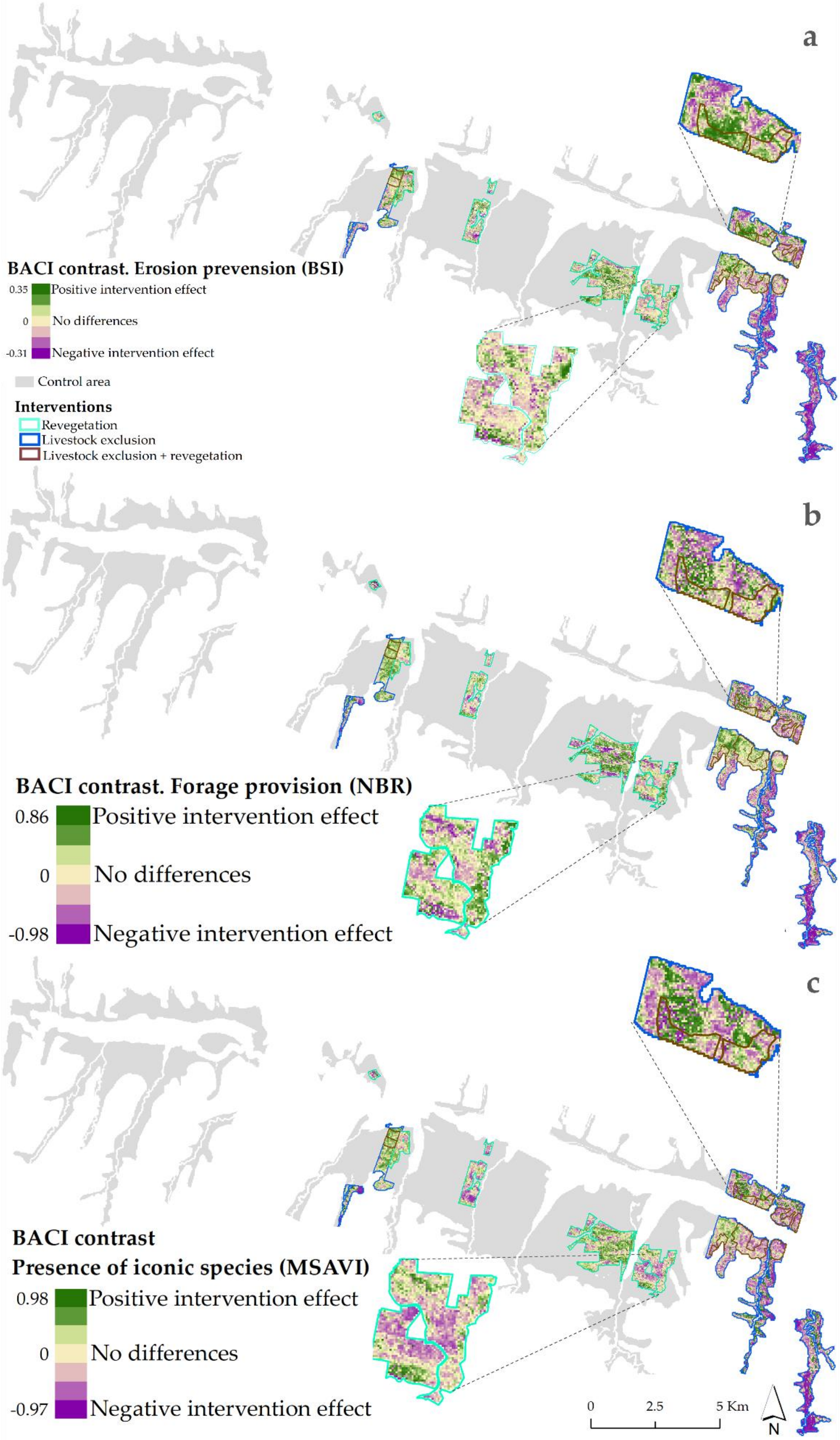

Figure 3. BACI contrast at pixel-level to assess the effect of each restoration intervention in the study area on: a) erosion prevention (based in the BSI index); b) forage provision (based on NBR index); and c) presence of iconic species (based on MSAVI index). The thicket and shrubland area used to for selecting control sites is indicated in grey.

3.3. Comparison of BACI contrast between interventions, vegetation clusters and terrain variables

In Table 6 the differences in BACI contrast between restoration interventions for the different ecosystem services are presented, as a measure of impact of these interventions. The interquartile range (IQR, the difference between the 25th and 75th percentile) is presented as indication of variability of the BACI contrast within these intervention sites. The lower this value, the more homogenous the intervention effect. The Hodges-Lehmann estimator of the median was used as the non-parametric indicator for each group [88], estimating a "pseudo–median" for non-symmetric populations, which is closely related to the population median [89].

The post-hoc test indicated that 'revegetation' sites are not significantly different from 'livestock exclusion + revegetation' sites. Both areas did not present evident impact on erosion prevention, while the BACI contrast for 'livestock exclusion' sites even showed a negative effect on this ecosystem service (Table 6). For forage provision, 'revegetation' areas performed significantly better (positive BACI contrast) as compared with the other two interventions. Whereas for the presence of iconic species, 'revegetation' sites did not show intervention impact while the other two interventions presented negative BACI contrasts, so decreased. The results in Table 6 show that at most sites across the three restoration interventions ecosystem services remained unchanged or decreased instead of the intended increase.

Table 6. Comparison of BACI contrast between interventions at pixel-level using the Kruskal-Wallis test (Games-Howell post-hoc). IQR = interquartile range

Landsat index	Represented ecosystem service	Intervention	Hodges-Lehmann estimator	IQR	Post-hoc*
BSI	Erosion prevention	Revegetation	0.00	0.08	a
		Livestock exclusion	0.06	0.13	b
		Livestock exclusion + revegetation	0.01	0.10	a
NBR	Forage provision	Revegetation	0.04	0.17	a
		Livestock exclusion	-0.11	0.16	b
		Livestock exclusion + revegetation	-0.02	0.17	c
MSAVI	Presence of iconic species	Revegetation	0.00	0.13	a
		Livestock exclusion	-0.06	0.19	b
		Livestock exclusion + revegetation	-0.04	0.11	b

*different letters indicate significant differences of Games-Howell post-hoc at p-value < 0.05

We also evaluated the relation between the state of the vegetation before interventions took place (as indicated by their cluster) and the BACI contrast. Vegetation clusters 1 and 2 comprised areas with relatively more vegetation, whereas vegetation clusters 4 and 5 contain more bare soil. No significant differences between clusters were found for 'revegetation' sites for erosion prevention; and better BACI contrast in vegetation clusters 4 and/or 5 for forage provision and presence of iconic species (Table 7). We excluded cluster 5 to compare 'revegetation' sites for the presence of iconic species because the area was too small resulting in insufficient independent pixels to run the comparison. Among all the evaluated restoration interventions sites, the variability of forage provision in cluster 1 (expressed by the IQR) was considerably higher than for the other clusters. This cluster represents 4.2% of the total intervened pixels. For 'livestock exclusion' sites, the contrasts for erosion prevention showed a negative impact (as shown by a positive Hodges-Lehmann estimator value) in clusters representing denser vegetation (i.e. clusters 1-3) before the intervention began. Forage provision only showed positive effect at 'livestock exclusion' sites in vegetation cluster 5 (with originally little vegetation), showing significant differences with clusters 2, 3 and 4. Presence of iconic species did not show a clear difference between clusters, while cluster 2 presented the only positive or neutral BACI contrasts for livestock exclusion' and 'livestock exclusion + revegetation' sites. From Table 7 we can observe the general tendency that especially areas which had little vegetation before the restoration intervention (Cluster 4-5), show a small, but significant increase in ecosystem services.

Regarding the assessed terrain variables to explain the intervention impact we found that south-facing slopes showed significantly better BACI contrast for presence of iconic species. In contrast, north-facing slopes presented a positive effect on forage provision (details are presented in Tables S2). The comparison of parent material classes showed consistent negative effects of the interventions across all ecosystem services for Quartzitic sandstone (shale). This parent material represents an average of 51% of all pixels. Regression models between BACI contrast with elevation and slope did not show any clear association, with R^2 lower than 0.06 for all ecosystem services and interventions (Table S5).

Table 7. Comparison of BACI contrast between vegetation clusters indicating the state of the vegetation for each intervention and ecosystem service using the Kruskal-Wallis test (Games-Howell post-hoc). NSD = No significant differences between groups. IQR = interquartile range. Cluster numbers are ordered from initial high to low vegetation cover (e.g. cluster 1 = denser vegetation).

Intervention	Landsat index	Represented ecosystem service	Cluster	Hodges-Lehmann estimator	IQR	Post hoc*
Revegetation	BSI	Erosion prevention	1	-0.02	0.19	NSD
			2	-0.04	0.10	
			3	0.00	0.10	
			4	0.00	0.09	
			5	0.00	0.06	
	NBR	Forage provision	1	0.08	0.47	ab
			2	-0.16	0.16	a
			3	-0.06	0.21	a
			4	0.02	0.18	b
			5	0.02	0.13	b
	MSAVI	Presence of iconic species	1	-0.01	0.21	a
			2	0.00	0.13	a
			3	-0.01	0.14	a
			4	0.05	0.12	b
Livestock exclusion	BSI	Erosion prevention	1	0.09	0.06	a
			2	0.07	0.10	a
			3	0.04	0.12	ab
			4	-0.01	0.09	c
			5	0.00	0.06	bc
	NBR	Forage provision	1	-0.01	0.39	abc
			2	-0.01	0.13	a
			3	-0.11	0.16	a
			4	-0.04	0.16	c
			5	0.06	0.04	b
	MSAVI	Presence of iconic species	1	-0.09	0.27	ab
			2	0.04	0.13	c
			3	-0.01	0.13	d
			4	-0.01	0.19	bd
			5	-0.07	0.05	a
Livestock exclusion + revegetation	BSI	Erosion prevention	1	-0.01	0.15	a
			2	0.01	0.12	ab
			3	0.04	0.06	b
			4	-0.01	0.06	a
			5	0.00	0.08	a
	NBR	Forage provision	1	0.02	0.30	ab
			2	-0.08	0.20	b
			3	-0.01	0.16	a
			4	0.00	0.12	a
			5	0.02	0.10	a
	MSAVI	Presence of iconic species	1	-0.11	0.25	a
			2	0.00	0.13	b
			3	-0.05	0.04	a
			4	-0.06	0.04	a
			5	-0.07	0.03	a

*different letters indicate significant differences of Games-Howell post-hoc at p-value < 0.05

4. Discussion

4.1. Selection of spectral indices

Our RS-based models suggest that spectral indices extracted from Landsat images can help to quantify the supply of the studied ecosystem services in the region. The indices that best captured the ecosystem services in the study area are based on the blue, red, near-infrared (NIR), and short-wave infrared (SWIR 1) wavelengths. Since we used the stratified vegetation cover as erosion prevention indicator, the BSI could effectively reflect the lack of this cover. Besides the BSI, the NBR index also presented a good fit with erosion prevention. Although NBR is usually used to detect burned areas, the index (NIR − SWIR)/(NIR + SWIR) has been referred to with different terminology [83] and as such has been also used to estimate wet and dry biomass [90], forest harvest [91], gross primary production [92] and vegetation water content [93].

Even though the presence of iconic species is linked to the percentage of spekboom cover, the model did not capture the presence of one single species as precisely (53% of the spekboom cover variation) as models that include the overall presence of green vegetation (StrVC and green biomass). In agreement with [50], the integration of elevation improved the capacity of the RS-model to capture the presence of iconic species, explaining 22% of the variance not captured by the Landsat 8 index. The model suggests that the presence of spekboom increases with elevation within the intervened area (between 380 and 680 meter above sea level). Among other reasons, elevations of 390 meter and lower are on the frost-prone valley floor and could impair the growth of spekboom [56].

The estimation of RS-based ecosystem services using field measurements provide higher accuracy and lower site-specific errors than estimations based on individual land cover types [49,94]. However, temporal extrapolation of such models with RS indices requires validation with data across time to decrease the uncertainty in the evaluation of restoration interventions. In this study, we tested our fitted models with other images acquired during the same fieldwork period. However, we could not validate the stability of the relationship between the spectral images and ecosystem services in the field for the full 30-year period.

4.2. RS-based BACI analyses

The BACI approach allows for relative comparisons of spatial and temporal differences that can be used to extract the unbiased restoration impact [55,95]. However, correct understanding of the underlying calculation process is needed to accurately interpret results. First, for each restoration intervention, BACI was applied using a different number of years for the pre-intervention period (Table S1). This will affect the BACI contrast, given that the spectral indices values vary between years [32], and consequently the result of the BACI contrast (i.e. the impact of restoration interventions) are also affected by the specific conditions of that selected year. For example, droughts or rainy years will affect vegetation cover and therefore the values of spectral indices. Assuming that climatic conditions are rather homogeneous in the neighborhood of the restored sites, this problem was partially solved by comparing the conditions of the restoration area before and after the intervention with those of similar areas nearby. Also, taking more than one year into account for the period 'before' and 'after' of the BACI calculations helps to compensate for index inter-annual variation, because it ensures that coincidental temporal variations do not restrict the identification of the effects [54]. Secondly, each pixel is assigned a p-value that shows the significance of the BACI contrast using 20 control sites (e.g. Figure S8). We compared 20 control pixels for each intervened pixel, and it is important to consider that those non-significant BACI contrasts can become significant when increasing the number of controls to, for example,100 pixels. However, this calculation would be more time and computational consuming and our explorative analysis with 100 control sites resulted in similar interventions impacts.

The pixel-level implementation of BACI using RS data assists in better spatially explicit evaluations of restoration interventions. Our method is particularly efficient for collecting historical data and evaluating large, remote, and heterogeneous areas where data collection is difficult and resource consuming. Our study area is located in a dry area with relatively little cloud cover. The availability of RS images will decrease in areas with frequent cloudy days, such as the in humid tropics. Depending on the availability of satellite images, the 'before' and 'after' reference periods could be changed or extended, allowing, for example, for the exclusion of abnormally dry years, or adding

another after period to evaluate the differences at different intervals of times from the start of the intervention.

Although the levels of the BACI contrast are small when expressed as absolute numbers (e.g. forage provision ranging from 0.04 to -0.11 in Table 6, translating to -9 to 11 kg per m^2) they can represent high relative change in ecosystem supply. The relative BACI contrast (as presented in Figure S7) highlights the magnitude of the contrast in relation to the analyzed pixel's spectral index value before the intervention took place. The percentages in the map of Figure S7 are particularly high when the baseline spectral value was close to zero.

4.3. Comparison of BACI contrast between interventions, vegetation clusters and terrain variables

Our presented pixel-level restoration evaluation method is specifically useful to evaluate heterogeneous landscapes, where restoration impact not only varies between restoration sites but also within. The inclusion of several control sites, a multi-year period 'before' in the BACI analyses, and its comparison with the current state allowed capturing the spatial and temporal effects of interventions otherwise invisible. Contrary to our initial assumption, we learned for example that livestock exclusion showed on average a negative impact compared to control sites and to 30 years ago. The maps in Section 4.2 visualize how this impact is spatially distributed within the intervened sites in order to guide future management of the area. The inclusion of vegetation clusters representing the status of vegetation before the intervention began, allowed to visualize if areas that originally had more vegetation responded better or worse than areas having little vegetation. Although the provision of ecosystem services in scarcely vegetated areas is low (e.g. average green biomass of 11.4 kg m^{-2}), in several cases these locations presented a positive effect of restoration interventions, especially in areas under revegetation. In contrast, areas that were originally more densely vegetated and still show high vegetation growth, showed negative effects of the interventions. The better BACI contrasts of less vegetated areas could be explained by considering that when the baseline supply of an ecosystem service is so small (e.g. stratified vegetation cover of 1%), any improvement change will reflect as a great difference. In contrast,

assessments have found that more severely degraded areas have lower restoration successes [96]. In our study we could not compare the BACI contrast to a quantified target value the restorations interventions aimed that. While we observed changes ecosystem services supply, without a target value we cannot make statements of intervention success. Regardless, more research would be needed to confirm this behavior and understand if there are other reasons of why originally vegetated areas responded more poorly to interventions than areas having little vegetation.

The inclusion of aspect and parent material allowed capturing differences and gaining insights for the interpretation of intervention impacts and potentially guiding intervention actions. Depending on the context of the interventions and specific monitoring objectives, the BACI contrast comparison could be improved by including other variables in the analyses. For example, by aspect and parent material of each intervention; year of when the revegetation started (from 2010 to 2015), or the inclusion of other fine scale data (e.g. high-resolution historical climate data). Variables correlated with land use intensity and past land use trends, may also influence spatial heterogeneity of the restoration effect [97].

5. Conclusions

The new evaluation method presented in this paper allows for mapping and quantifying the long-term impact of restoration interventions on ecosystem services at pixel-level. Our approach helps to differentiate the intervention effect from other environmental factors and is especially useful for monitoring large, remote, and heterogeneous landscapes. The resulting maps visualize spatial patterns of intervention effects within larger intervention sites, and provide insights on their temporal changes As such, these maps help to learn from restoration experience and mitigate ineffective future restoration efforts. Moreover, our approach allows us to identify which spatially-variable factors may explain the success or failure of an intervention. By measuring restoration impact on different ecosystem services, we increase our understanding of social benefits and trade-offs of restoration choices. The presented approach can be extended to a broader range of restoration interventions and ecosystem services in different contexts across different landscapes, as long as spectral indices and spatial indicators can be identified to represent these ecosystem services. Our RS-based approach is particularly suitable to

estimate changes in ecosystem services directly linked with the presence of vegetation and less of ecosystem services that are strongly linked to a specific species or human perception, for which additional spatial data would be needed. Our understanding of the well RS-based ecosystem service models perform over multi-year periods can be improved by having long-term research sites to validate relations between field observations and spectral information over time.

Acknowledgments

We are grateful to members of Living Lands in the Baviaanskloof Hartland Bawarea Conservancy, South Africa for support in providing network and logistic facilitation, providing crucial background data, assistance for fieldwork facilitation (especially from Elwin Malgas, Luyanda Luthuli, Melloson Allen, and the interns Jurian Schepers and Amanda Alfonso-Herrera), providing working facilities and the friendly and enabling environment. We extend our appreciation to Michele Meroni, for clarifying methods to carry out the analyses of intervention restorations. Finally, we would like to thank all the farmers involved for their cooperation, friendliness, accessibility and knowledge sharing during fieldwork.

References

1. Power AG. Ecosystem services and agriculture: tradeoffs and synergies. Philos Trans R Soc Lond B Biol Sci. 2010;365(1554):2959–71.

2. DeClerck FAJ, Jones SK, Attwood S, Bossio D, Girvetz E, Chaplin-Kramer B, et al. Agricultural ecosystems and their services: the vanguard of sustainability? Curr Opin Environ Sustain. 2016;23:92–9.

3. IPBES. Summary for policymakers of the assessment report on land degradation and restoration of the Intergovernmental Science- Policy Platform on Biodiversity and Ecosystem Services. R. Scholes, L. Montanarella, A. Brainich, N. Barger, B. ten Brink MC, B. Erasmus, J. Fisher, T. Gardner, T. G. Holland, F. Kohler, J. S. Kotiaho, G. Von Maltitz, G. Nangendo, R. Pandit, J. Parrotta MDP, S. Prince MS and LW, editors. IPBES secretariat, Bonn, Germany; 2018. 44 p.

4. Costanza R, de Groot R, Sutton P, van der Ploeg S, Anderson SJ, Kubiszewski I, et al. Changes in the global value of ecosystem services. Glob Environ Chang. 2014;26:152–8.

5. Pacheco FAL, Sanches Fernandes LF, Valle Junior RF, Valera CA, Pissarra TCT. Land degradation: Multiple environmental consequences and routes to neutrality. Curr Opin Environ Sci Heal. 2018;5:79–86.

6. Sutton PC, Anderson SJ, Costanza R, Kubiszewski I. The ecological economics of land degradation: Impacts on ecosystem service values. Ecol Econ. 2016;129:182–92.

7. Chazdon RL, Brancalion PHS, Lamb D, Laestadius L, Calmon M, Kumar C. A Policy-Driven Knowledge Agenda for Global Forest and Landscape Restoration. Conserv Lett. 2017;10(1):125–32.

8. United Nations. Conference of the Parties Convention to Combat Desertification. 2030 Agenda Sustain Dev Implic United Nations Conv to Combat Desertif. 2017;

9. Leadley, Paul W and Krug, Cornelia B and Alkemade, Rob and Pereira, Henrique M and Sumaila, U Rashid and Walpole, Matt and Marques, Alexandra and Newbold, Tim and Teh, L SL and van Kolck J and others. Progress towards the Aichi Biodiversity Targets: an assessment of biodiversity trends, policy scenarios and key actions, CBD Technical Series No. 78. Montreal, Canada; 2014.

10. Lal R, Safriel U, Boer B. Zero net land degradation: A new sustainable development goal for Rio+ 20. In: United Nations Convention to Combat Desertification (UNCCD). 2012.

11. ICSU. Sustainable Development Goals and targets. Int Counc Sci. 2015;

12. Programme UNE. Bonn Challenge. Bonn Challenge. 2011.

13. United Nations. New York Declaration on Forests: Action Statements and Action Plans. Clim Summit 2014. 2014;

14. Herrick J, Abrahamse T, Abhilash P, Ali S, Alvarez-Torres P, Barau A, et al. Land Restoration for Achieving the Sustainable Development Goals An International Resource Panel Think Piece. United Nations Environment Programme; 2019.

15. United Nations Environment Agency. New UN Decade on Ecosystem Restoration offers unparalleled opportunity for job creation, food security and addressing climate change. Press Release: Ecosystems and Biodiversity. Available from: https://www.unenvironment.org/news-and-stories/press-release/new-un-decade-ecosystem-restoration-offers-unparalleled-opportunity. 2019.

16. Cooke SJ, Bennett JR, Jones HP. We have a long way to go if we want to realize the promise of the "Decade on Ecosystem Restoration." Conserv Sci Pract. 2019;1(12):e129.

17. Fisher, J., Montanarella, L., and Scholes R. Chapter 1: Benefits to people from avoiding land degradation and restoring degraded land. In: In IPBES (2018): The IPBES assessment report on land degradation and restoration Montanarella, L, Scholes, R, and Brainich, A (eds) Secretariat of the Intergovernmental Science-Policy Platform on Biodiversity and Ecosystem Services. Bonn, Germany; 2018. p. 1–51.

18. Chazdon RL, Bodin B, Guariguata M, Lamb D, Walder B, Unna C, et al. Partnering with nature: the case

for natural regeneration in for-est and landscape restoration. FERI Policy Brief, Montreal Canada. Montreal, Canada: Forest Ecosystem Restoration Initiative; 2017.

19. Sattler D, Raedig C, Hebner A, Wesenberg J. Use of Native Plant Species for Ecological Restoration and Rehabilitation Measures in Southeast Brazil. In: Strategies and tools for a sustainable rural Rio de Janeiro. Springer; 2019. p. 191–204.

20. Castillo-Escrivà A, López-Iborra GM, Cortina J, Tormo J. The use of branch piles to assist in the restoration of degraded semiarid steppes. Restor Ecol. 2019;27(1):102–8.

21. Prach K, Hobbs RJ. Spontaneous succession versus technical reclamation in the restoration of disturbed sites. Restor Ecol. 2008;16(3):363–6.

22. Zhang J, Luo M, Cao S. How deep is China's environmental Kuznets curve? An analysis based on ecological restoration under the Grain for Green program. Land use policy. 2018;70:647–53.

23. Jordan F, Arrington DA. Piscivore Responses to Enhancement of the Channelized Kissimmee River, Florida, U.S.A. Restor Ecol. 2014;22(3):418–25.

24. Powers SP, Peterson CH, Grabowski JH, Lenihan HS. Success of constructed oyster reefs in no-harvest sanctuaries: Implications for restoration. Mar Ecol Prog Ser. 2009;389:159–70.

25. Keenleyside K, Dudley N, Cairns S, Hall C, Stolton S. Ecological restoration for protected areas: Principles, guidelines and best practices. Best Practice Protected Area Guidelines Series. Gland, Switzerland: IUCN; 2012. 120 p.

26. Wortley L, Hero JM, Howes M. Evaluating ecological restoration success: A review of the literature. Restor Ecol. 2013;21(5):537–43.

27. Willemen L, Barger NN, Brink B ten, Cantele M, Erasmus BFN, Fisher JL, et al. How to halt the global decline of lands. Nat Sustain. 2020;3(3):164–6.

28. Nilsson C, Aradottir AL, Hagen D, Halldórsson G, Høegh K, Mitchell RJ, et al. Evaluating the process of ecological restoration. Ecol Soc. 2016;21(1).

29. Nunes A, Oliveira G, Mexia T, Valdecantos A, Zucca C, Costantini EAC, et al. Ecological restoration across the Mediterranean Basin as viewed by practitioners. Sci Total Environ. 2016;566:722–32.

30. Alexander S, Aronson J, Whaley O, Lamb D. The relationship between ecological restoration and the ecosystem services concept. Ecol Soc. 2016;21(1).

31. Bullock JM, Aronson J, Newton AC, Pywell RF, Rey-Benayas JM. Restoration of ecosystem services and biodiversity: Conflicts and opportunities. Trends Ecol Evol. 2011;26(10):541–9.

32. Meroni M, Schucknecht A, Fasbender D, Rembold F, Fava F, Mauclaire M, et al. Remote sensing monitoring of land restoration interventions in semi-arid environments with a before–after control-impact statistical design. Int J Appl Earth Obs Geoinf. 2017;59:42–52.

33. De Araujo Barbosa CC, Atkinson PM, Dearing JA. Remote sensing of ecosystem services: A systematic review. Ecol Indic. 2015;52:430–43.

34. Skidmore AK, Pettorelli N, Coops NC, Geller GN, Hansen M, Lucas R, et al. Environmental science: Agree on biodiversity metrics to track from space. Nature. 2015;523(7561):403–5.

35. Cord AF, Brauman KA, Chaplin-Kramer R, Huth A, Ziv G, Seppelt R. Priorities to Advance Monitoring of Ecosystem Services Using Earth Observation. Trends Ecol Evol. 2017;32(6):416–28.

36. Verbesselt J, Hyndman R, Newnham G, Culvenor D. Detecting trend and seasonal changes in satellite image time series. Remote Sens Environ. 2010;114(1):106–15.

37. Rieb JT, Chaplin-Kramer R, Daily GC, Armsworth PR, Böhning-Gaese K, Bonn A, et al. When, Where, and How Nature Matters for Ecosystem Services: Challenges for the Next Generation of Ecosystem Service Models. Bioscience. 2017;67(9):820--833.

38. Wu W. Derivation of tree canopy cover by multiscale remote sensing approach. ISPRS-International Arch Photogramm Remote Sens Spat Inf Sci. 2011;38(4):142–9.

39. Pandit S, Tsuyuki S, Dube T. Estimating above-ground biomass in sub-tropical buffer zone community forests, Nepal, using Sentinel 2 data. Remote Sens. 2018;10(4).

40. Wang Z, Bastin GN, Liu L, Caccetta PA, Peng D. Estimating woody above-ground biomass in an arid zone of central Australia using Landsat imagery. J Appl Remote Sens. 2015;9(1):096036.

41. Frampton WJ, Dash J, Watmough G, Milton EJ. Evaluating the capabilities of Sentinel-2 for quantitative estimation of biophysical variables in vegetation. ISPRS J Photogramm Remote Sens. 2013;82:83–92.

42. Willemen L. It's about time: Advancing spatial analyses of ecosystem services and their application. Ecosyst Serv. 2020;44:101125.

43. Pasquarella VJ, Holden CE, Kaufman L, Woodcock CE. From imagery to ecology: leveraging time series of all available Landsat observations to map and monitor ecosystem state and dynamics. Remote Sens Ecol Conserv. 2016;2(3):152–70.

44. Li W, Gong P. Continuous monitoring of coastline dynamics in western Florida with a 30-year time series of Landsat imagery. Remote Sens Environ. 2016;179:196--209.

45. del Río-Mena T, Willemen L, Vrieling A, Nelson A. Understanding intra-annual dynamics of ecosystem services using satellite image time series. Remote Sens. 2020;12(4):710.

46. Ramirez-Reyes C, Brauman KA, Chaplin-Kramer R, Galford GL, Adamo SB, Anderson CB, et al. Reimagining the potential of Earth observations for ecosystem service assessments. Sci Total Environ. 2019;665(2019):1053--1063.

47. Tallis H, Mooney H, Andelman S, Balvanera P, Cramer W, Karp D, et al. A Global System for Monitoring Ecosystem Service Change. Bioscience. 2012;62(11):977–86.

48. Jetz W, Cavender-Bares J, Pavlick R, Schimel D, Davis FW, Asner GP, et al. Monitoring plant functional diversity from space. Nat Plants. 2016;2(3):1–5.

49. Eigenbrod F, Armsworth PR, Anderson BJ, Heinemeyer A, Gillings S, Roy DB, et al. The impact of proxy-based methods on mapping the distribution of ecosystem services. J Appl Ecol. 2010;47(2):377–85.

50. Martínez-Harms MJ, Quijas S, Merenlender AM, Balvanera P. Enhancing ecosystem services maps combining field and environmental data. Ecosyst Serv. 2016;22:32–40.

51. Green RH. Sampling Design and Statistical Methods for Environmental Biologists. New York, NY (USA): Wiley-Interscience Pub.; 1979.

52. Underwood AJ. On beyond BACI: Sampling designs that might reliably detect environmental disturbances. Ecol Appl. 1994;4(1):3–15.

53. Zucca C, Wu W, Dessena L, Mulas M. Assessing the Effectiveness of Land Restoration Interventions in Dry Lands by Multitemporal Remote Sensing - A Case Study in Ouled DLIM (Marrakech, Morocco). L Degrad Dev. 2015;26(1):80–91.

54. Underwood AJ. Beyond BACI: the detection of environmental impacts on populations in the real, but variable, world. J Exp Mar Bio Ecol. 1992;161(2):145–78.

55. Schwarz CJ. Analysis of BACI experiments. In Course Notes for Beginning and Intermediate Statistics. Available from: http://www.stat.sfu.ca/~cschwarz/CourseNotes. 2019. p. 08–20.

56. Duker R, Cowling RM, du Preez DR, Potts AJ. Frost, Portulacaria afra Jacq., and the boundary between the Albany Subtropical Thicket and Nama-Karoo biomes. South African J Bot. 2015;101:112–9.

57. Bochet E, García-Fayos P, Poesen J. Topographic thresholds for plant colonization on semi-arid eroded slopes. Earth Surf Process Landforms. 2009;34(13):1758–71.

58. Måren IE, Karki S, Prajapati C, Yadav RK, Shrestha BB. Facing north or south: Does slope aspect impact

forest stand characteristics and soil properties in a semiarid trans-Himalayan valley? J Arid Environ. 2015;121:112–23.

59. Kutiel P, Lavee H. Effect of slope aspect on soil and vegetation properties along an aridity transect. Isr J Plant Sci. 1999;47(3):169–78.

60. Anderson DW. The effect of parent material and soil development on nutrient cycling in temperate ecosystems. Biogeochemistry. 1988;5(1):71–97.

61. WRC. Water Research Commission. Annual Report. Pretoria, South Africa: WRC; 2018.

62. Van Luijk G, Cowling RM, Riksen MJPM, Glenday J. Hydrological implications of desertification: Degradation of South African semi-arid subtropical thicket. J Arid Environ. 2013;91:14–21.

63. Vlok JHJ, Euston-Brown DIW, Cowling RM. Acocks' Valley Bushveld 50 years on: New perspectives on the delimitation, characterisation and origin of subtropical thicket vegetation. South African J Bot. 2003;69(1):27–51.

64. Havstad KM, Herrick JE, Schlesinger WH. Desert rangelands, degradation and nutrients. In: Rangeland Desertification. Dordrecht: Springer Netherlands; 2000. p. 77–87.

65. Marais C, Cowling R, Powell M, Mills A. Establishing the platform for a carbon sequestration market in South Africa: The Working for Woodlands Subtropical Thicket Restoration Programme. In: XIII World Forestry Congress, Buenos Aires. 2009. p. 18–23.

66. Stuart-Hill GC. Effects of Elephants and Goats on the Kaffrarian Succulent Thicket of the Eastern Cape, South Africa. J Appl Ecol. 1992;699–710.

67. Lechmere-Oertel RG, Kerley GIH, Cowling RM. Patterns and implications of transformation in semi-arid succulent thicket, South Africa. J Arid Environ. 2005;62(3):459–74.

68. Sigwela AM, Kerley GIH, Mills AJ, Cowling RM. The impact of browsing-induced degradation on the reproduction of subtropical thicket canopy shrubs and trees. South African J Bot. 2009;75(2):262–7.

69. van Eck B, Selomane O, van den Broeck D, Zylstra M, de Groot RS. Cultivating the 'PRESENCE' Learning Network to Restore Living Landscapes: Adapting to Climate Change in the Baviaanskloof Catchment, South Africa. Build Resil to Clim Chang Ecosyst Adapt lessons from F. 2010;135–50.

70. Mills A, Fey M. Transformation of thicket to savanna reduces soil quality in the Eastern Cape, South Africa. Plant Soil. 2004;265(1–2):153–63.

71. Mills AJ, Turpie JK, Cowling RM, Marais C, Kerley GIH, Lechmere-Oertel RG, et al. Assessing costs, benefits, and feasibility of restoring natural capital in Subtropical Thicket in South Africa. Restoring Nat Cap Sci Bus Pract (The Sci Pract Ecol Restor Ser. 2007;2:179–87.

72. Mills AJ, Robson A. Survivorship of spekboom (Portulacaria afra) planted within the subtropical thicket restoration programme. S Afr J Sci. 2017;113(1–2):3–5.

73. Powell MJ. Restoration of degraded subtropical thickets in the Baviaanskloof Megareserve. Africa (Lond). 2009;(June):1–151.

74. Van der Vyver ML, Cowling RM, Mills AJ, Difford M. Spontaneous return of biodiversity in restored subtropical thicket: Portulacaria afra as an ecosystem engineer. Restor Ecol. 2013;21(6):736–44.

75. Zhongming W, Lees BG, Feng J, Wanning L, Haijing S. Stratified vegetation cover index: A new way to assess vegetation impact on soil erosion. Catena. 2010;83(1):87–93.

76. Flombaum P, Sala OE. A non-destructive and rapid method to estimate biomass and aboveground net primary production in arid environments. J Arid Environ. 2007;69:352–8.

77. del Río-Mena T, Willemen L, Vrieling A, Nelson A. Remote sensing for mapping ecosystem services to support evaluation of ecological restoration interventions in an arid landscape. Ecol Indic. 2020;113(May 2019):106182.

78. Kock N, Lynn GS. Lateral Collinearity and Misleading Results in Variance-Based SEM : An Illustration and Recommendations. J Assoc Inf Syst. 2012;13(7):546–80.

79. Kuhn M. The caret Package: Classification and Regression Training. R Found Stat Comput Vienna, Austria http//topepo.github.io/caret/index.html. 2019;

80. Akaike H. A New Look at the Statistical Model Identification. IEEE Trans Automat Contr. 1974;19(6):716–23.

81. Bartón K. MuMIn: Multi-Model Inference. R package version 1.40.4. https://CRAN.R-project.org/. 2018.

82. Efron B, Gong G. A leisurely look at the bootstrap, the jackknife, and cross-validation. Am Stat. 1983;37(1):36–48.

83. Ji L, Zhang L, Wylie BK, Rover J. On the terminology of the spectral vegetation index (NIR- SWIR)/(NIR+ SWIR). Int J Remote Sens. 2011;32(21):6901--6909.

84. USGS. Landsat Collection 1 Surface Reflectance. Available from: https://www.usgs.gov/core-science-systems/nli/landsat/landsat-collection-1-surface-reflectance. 2020.

85. Geophysical Institute of the University of Alaska Fairbanks. Alaska Satellite Facility (ASF) Data Portal. Available from: https://search.asf.alaska.edu/#/. 2018.

86. (CGS). South African Council for Geoscience. Available from: https://geoscience.org.za/cgs/. 2018.

87. De Oto L, Vrieling A, Fava F, de Bie K (C AJM). Exploring improvements to the design of an operational seasonal forage scarcity index from NDVI time series for livestock insurance in East Africa. Int J Appl Earth Obs Geoinf. 2019;82:101885.

88. Hodges JL, Lehmann EL. Estimates of Location Based on Rank Tests. Ann Math Stat. 1963;598–611.

89. Hettmansperger TP, McKean JW. Robust nonparametric statistical methods. Second Edition. CRC Press; 2010.

90. Kimes DS, Markham BL, Tucker CJ, McMurtrey JE. Temporal relationships between spectral response and agronomic variables of a corn canopy. Remote Sens Environ. 1981;11:401–11.

91. Wilson EH, Sader SA. Detection of forest harvest type using multiple dates of Landsat TM imagery. Remote Sens Environ. 2002;80(3):385–96.

92. Xiao X, Hollinger D, Aber J, Goltz M, Davidson EA, Zhang Q, et al. Satellite-based modeling of gross primary production in an evergreen needleleaf forest. Remote Sens Environ. 2004;89(4):519–34.

93. Chen D, Huang J, Jackson TJ. Vegetation water content estimation for corn and soybeans using spectral indices derived from MODIS near- and short-wave infrared bands. Remote Sens Environ. 2005;98(2–3):225–36.

94. Lavorel S, Grigulis K, Lamarque P, Colace MP, Garden D, Girel J, et al. Using plant functional traits to understand the landscape distribution of multiple ecosystem services. J Ecol. 2011;99(1):135–47.

95. Smith EP. BACI Design. El-Shaarawi AH, Piegorsch WW, editors. Encycl Environmetrics. 2014;1:141–8.

96. Pandit, R., Parrota, J., Anker, Y., Coudel E, Diaz Morejón, C. F., Harris, J., Karlen DL, Kertész, Á. MDPJL, Ntshotsho Simelane, P., Tamin, N. M. A, Vieira DLM. Chapter 6: Responses to halt land degradation and to restore degraded land. In: IPBES (2018): The IPBES assessment report on land degradation and restoration Montanarella, L, Scholes, R, and Brainich, A (eds) Secretariat of the Intergovernmental Science-Policy Platform on Biodiversity and Ecosystem Services. Bonn, Germany; 2018. p. 435–528.

97. Maass JM, Balvanera P, Castillo A, Daily GC, Mooney HA, Ehrlich P, et al. Ecosystem services of tropical dry forests: Insights from long-term ecological and social research on the Pacific Coast of Mexico. Ecol Soc. 2005;10(1):1–23.

Click here to access/download
Supporting Information
SuplementaryMaterials_BACI_Final.docx
